# Supplementary material for: β-catenin-promoted cholesterol metabolism protects against cellular senescence in naked mole-rat cells
Source: Commun Biol. 2021 Mar 19;4:357. doi: 10.1038/s42003-021-01879-8 (PMC7979689; doi:10.1038/s42003-021-01879-8)

Supplementary information

β-catenin-promoted cholesterol metabolism protects against cellular senescence in naked mole-rat cells

Woei-Yaw Chee, Yuriko Kurahashi, Junhyeong Kim, Kyoko Miura, Daisuke Okuzaki, Tohru Ishitani, Kentaro Kajiwara, Shigeyuki Nada, Hideyuki Okano and Masato Okada

Contents:

Supplementary Tables 1-4
Supplementary Figures 1-16
Original Immunoblot/gel images in figures; 6 pages

Supplementary Table 1. Representative downregulated genes within DEG from RNA-seq

Fold Change	gene name	Description
-13.584	Wnt10b	Wnt family member 10B
-12.199	Lpar3	lysophosphatidic acid receptor 3
-11.079	LOC110345950	uncharacterized LOC110345950
-10.307	Fam220a	family with sequence similarity 220 member A
-8.317	LOC101716611	uncharacterized LOC101716611
-7.796	Eln	elastin
-7.686	Ctnnb1	catenin beta 1
-7.656	Mxi1	"MAX interactor 1, dimerization protein"
-7.334	Cavin2	caveolae associated protein 2
-6.376	Cpxm1	"carboxypeptidase X, M14 family member 1"
-6.322	Fam198a	Golgi Associated Kinase 1A
-6.191	Efhd1	EF-hand domain family member D1
-5.845	LOC110348574	uncharacterized LOC110348574
-5.826	Entpd3	ectonucleoside triphosphate diphosphohydrolase 3
-5.822	LOC101725231	60S ribosomal protein L9 pseudogene
-5.822	LOC110346661	uncharacterized LOC110346661
-5.822	LOC106010386	uncharacterized LOC106010386
-5.822	LOC101710023	uncharacterized LOC101710023
-5.822	LOC106008187	uncharacterized LOC106008187
-5.813	Trerf1	transcriptional regulating factor 1
-5.715	Ano1	anoctamin 1
-5.452	Dlk2	delta like non-canonical Notch ligand 2
-5.446	Ankrd29	ankyrin repeat domain 29
-5.406	Sox2	SRY-box 2
-5.120	Egfl6	EGF like domain multiple 6
-4.991	LOC110348191	uncharacterized LOC110348191
-4.990	LOC101717878	myc-associated zinc finger protein pseudogene
-4.990	LOC110346939	zinc finger protein 670 pseudogene
-4.990	LOC110346980	gametocyte-specific factor 1 pseudogene
-4.990	LOC110350373	uncharacterized LOC110350373
-4.990	LOC101711524	transcriptional coactivator YAP1 pseudogene
-4.990	LOC110347540	uncharacterized LOC110347540
-4.981	Efcab1	EF-hand calcium binding domain 1
-4.713	Pi15	peptidase inhibitor 15
-4.671	Map2k6	mitogen-activated protein kinase kinase 6
-4.614	Morn5	MORN repeat containing 5
-4.574	LOC110350023	uncharacterized LOC110350023
-4.574	LOC101718238	protein AF-9-like
-4.574	LOC110348851	uncharacterized LOC110348851
-4.395	S1pr5	sphingosine-1-phosphate receptor 5
-4.366	LOC110345400	E3 ubiquitin-protein ligase HACE1-like
-4.348	Cgnl1	cingulin like 1
-4.347	LOC101712708	uncharacterized LOC101712708
-4.325	LOC101707839	cadherin-18
-4.286	LOC101704034	"UDP-GalNAc:beta-1,3-N-acetylgalactosaminyltransferase 1-like"
-4.282	Ctnna2	catenin alpha 2
-4.268	Fuca2	alpha-L-fucosidase 2
-4.256	Peg10	paternally expressed 10
-4.158	LOC106009933	ras-related protein Rab-5A-like
-4.158	Apof	apolipoprotein F
-4.158	LOC110347698	eukaryotic translation initiation factor 4E pseudogene
-4.158	LOC101712597	cyclin-dependent kinase 1 pseudogene
-4.158	LOC101718707	small integral membrane protein 19 pseudogene
-4.158	Mylpf	"myosin light chain, phosphorylatable, fast skeletal muscle"
-4.158	LOC106009357	vesicle transport protein GOT1B-like
-4.158	LOC101712679	40S ribosomal protein SA pseudogene
-4.158	LOC101707806	intercellular adhesion molecule 3
-4.158	CUNH10orf10	DEPP1 Autophagy regulator
-4.006	Serpinb7	serpin family B member 7

Supplementary Table 2. Representative upregulated genes within DEG from RNA-seq

Fold Change	gene name	Description
4.008	LOC101696494	UDP-glucuronosyltransferase 1-9-like
4.008	LOC101708467	U5 small nuclear ribonucleoprotein 40 kDa protein pseudogene
4.008	Rasd1	ras related dexamethasone induced 1
4.008	Gpr15	G protein-coupled receptor 15
4.014	Sycp1	synaptonemal complex protein 1
4.016	Prr15	proline rich 15
4.029	LOC110344797	uncharacterized LOC110344797
4.030	LOC101707377	alveolar macrophage chemotactic factor
4.033	LOC106008493	uncharacterized LOC106008493
4.058	LOC110345612	uncharacterized LOC110345612
4.070	Spock2	"SPARC (osteonectin), cwcv and kazal like domains proteoglycan 2"
4.133	Cldn34	claudin 34
4.162	Gabbr2	gamma-aminobutyric acid type B receptor subunit 2
4.169	Mest	mesoderm specific transcript
4.169	CUNH15orf48	chromosome unknown C15orf48 homolog
4.183	LOC110345861	uncharacterized LOC110345861
4.207	LOC110344733	uncharacterized LOC110344733
4.208	LOC110350140	uncharacterized LOC110350140
4.208	LOC101699425	hippocampus abundant transcript-like protein 1 pseudogene
4.208	Lyg1	lysozyme g1
4.212	Dfna5	Non-sybdromic hearing impariment protein 5
4.214	LOC110344645	uncharacterized LOC110344645
4.231	Mcf2l2	MCF.2 cell line derived transforming sequence-like 2
4.255	Scarf1	scavenger receptor class F member 1
4.303	Fam83e	family with sequence similarity 83 member E
4.316	Plg	plasminogen
4.329	LOC110345217	uncharacterized LOC110345217
4.335	LOC110344203	uncharacterized LOC110344203
4.348	LOC101698571	uncharacterized LOC101698571
4.358	LOC101701112	histone-lysine N-methyltransferase PRDM9
4.383	Gtsf1	gametocyte specific factor 1
4.391	LOC110345541	uncharacterized LOC110345541
4.409	LOC110347769	translation initiation factor IF-2-like
4.409	LOC110344201	uncharacterized LOC110344201
4.409	Podxl2	podocalyxin like 2
4.409	LOC101696562	uncharacterized LOC101696562
4.482	Zfyve28	zinc finger FYVE-type containing 28
4.486	LOC110349692	uncharacterized LOC110349692
4.493	LOC101706767	platelet basic protein-like
4.500	CUNH2orf81	chromosome unknown C2orf81 homolog
4.529	Sla	Src like adaptor
4.542	Rassf10	Ras association domain family member 10
4.609	Thy1	Thy-1 cell surface antigen
4.620	Cfap126	cilia and flagella associated protein 126
4.646	Msr1	macrophage scavenger receptor 1
4.659	Kcnn3	potassium calcium-activated channel subfamily N member 3
4.750	Tnip3	TNFAIP3 interacting protein 3
4.810	Kcnab1	potassium voltage-gated channel subfamily A member regulatory beta subunit 1
4.810	LOC110348315	uncharacterized LOC110348315
4.810	LOC110345795	uncharacterized LOC110345795
4.810	LOC110347917	uncharacterized LOC110347917
4.810	LOC101701625	nascent polypeptide-associated complex subunit alpha pseudogene
4.810	LOC110348910	uncharacterized LOC110348910
4.810	LOC101709453	growth hormone-inducible transmembrane protein pseudogene
4.810	LOC110344130	uncharacterized LOC110344130
4.810	LOC110347252	uncharacterized LOC110347252
4.810	LOC110349696	uncharacterized LOC110349696
4.810	LOC110347215	uncharacterized LOC110347215
4.810	Ptprh	protein tyrosine phosphatase receptor type H
4.810	LOC110349749	histone H4
4.810	LOC110343935	uncharacterized LOC110343935
4.810	LOC101721253	uncharacterized LOC101721253
4.810	Cspg5	chondroitin sulfate proteoglycan 5
4.813	LOC110346620	uncharacterized LOC110346620
4.854	LOC110347520	uncharacterized LOC110347520
4.954	LOC106007905	uncharacterized LOC106007905
5.017	LOC110350177	uncharacterized LOC110350177
5.034	Ehf	ETS homologous factor
5.091	Mmp8	matrix metallopeptidase 8
5.110	LOC110350464	uncharacterized LOC110350464
5.110	LOC101721336	uncharacterized LOC101721336
5.124	LOC101712048	serum amyloid A-4 protein
5.129	LOC106007907	uncharacterized LOC106007907
5.148	Izumo4	IZUMO family member 4
5.167	Prkch	protein kinase C eta
5.183	Tfec	transcription factor EC
5.186	LOC110350035	uncharacterized LOC110350035
5.210	LOC101711824	intersectin-2 pseudogene
5.210	LOC110349417	uncharacterized LOC110349417
5.210	LOC106010809	translationally-controlled tumor protein pseudogene
5.210	Plet1	placenta expressed transcript 1
5.215	LOC101723772	uncharacterized LOC101723772
5.221	LOC101725671	T-complex protein 1 subunit zeta
5.284	LOC110344690	uncharacterized LOC110344690
5.291	LOC106008440	uncharacterized LOC106008440
5.410	LOC110347960	uncharacterized LOC110347960
5.411	Ido1	"indoleamine 2,3-dioxygenase 1"
5.411	LOC110344930	uncharacterized LOC110344930
5.411	LOC101724224	uncharacterized LOC101724224
5.427	Scg2	secretogranin II
5.438	Gjb2	gap junction protein beta 2
5.475	LOC110344528	uncharacterized LOC110344528
5.581	Lancl3	LanC like 3
5.599	Hsd11b1	hydroxysteroid 11-beta dehydrogenase 1
5.611	LOC110348594	40S ribosomal protein S2 pseudogene
5.621	Hdac9	histone deacetylase 9
5.688	Cited1	Cbp/p300 interacting transactivator with Glu/Asp rich carboxy-terminal domain 1
5.711	LOC110347419	uncharacterized LOC110347419
5.772	LOC101698282	uncharacterized LOC101698282
5.789	Ptgs2	prostaglandin-endoperoxide synthase 2
5.877	Svep1	"sushi, von Willebrand factor type A, EGF and pentraxin domain containing 1"
6.012	LOC110348319	uncharacterized LOC110348319
6.012	Capns2	calpain small subunit 2
6.012	LOC101698865	eotaxin
6.012	LOC101701394	ADP-ribosylation factor-like protein 2-binding protein pseudogene
6.012	LOC110351010	histone H3
6.012	LOC110344276	uncharacterized LOC110344276
6.012	Foxa1	forkhead box A1
6.012	LOC101702235	ferritin light chain pseudogene
6.054	Hck	"HCK proto-oncogene, Src family tyrosine kinase"
6.105	Rgcc	regulator of cell cycle
6.168	Syn1	synapsin I
6.195	Acpp	"acid phosphatase, prostate"
6.456	Col28a1	collagen type XXVIII alpha 1 chain
6.613	Scg5	secretogranin V
6.633	Gjb6	gap junction protein beta 6
6.814	Cldn16	claudin 16
6.914	LOC110344990	uncharacterized LOC110344990
6.919	Olfm4	olfactomedin 4
7.214	LOC110344566	uncharacterized LOC110344566
7.214	LOC101715086	uncharacterized LOC101715086
7.214	LOC110346404	uncharacterized LOC110346404
7.214	LOC106009632	uncharacterized LOC106009632
7.214	LOC106010244	60S ribosomal protein L17 pseudogene
7.338	Pamr1	peptidase domain containing associated with muscle regeneration 1
7.522	Nrg2	neuregulin 2
7.666	Dcn	decorin
7.808	Ccdc141	coiled-coil domain containing 141
7.816	LOC101714691	uncharacterized LOC101714691
7.816	LOC110346356	uncharacterized LOC110346356
7.824	Cp	ceruloplasmin
8.237	LOC110348169	uncharacterized LOC110348169
8.331	LOC101705012	"HLA class II histocompatibility antigen, DRB1-4 beta chain"
8.417	Pdzk1ip1	PDZK1 interacting protein 1
8.417	LOC110348227	uncharacterized LOC110348227
8.417	LOC110344608	uncharacterized LOC110344608
8.417	LOC110350948	uncharacterized LOC110350948
8.417	LOC110350012	uncharacterized LOC110350012
8.417	LOC110346739	uncharacterized LOC110346739
9.379	LOC110344368	uncharacterized LOC110344368
9.619	LOC110345873	uncharacterized LOC110345873
9.619	LOC110345817	protein FAM177A1 pseudogene
9.672	Ccl20	C-C motif chemokine ligand 20
9.943	Rab11fip4	RAB11 family interacting protein 4
10.268	LOC101718744	plasminogen activator inhibitor 2
10.462	Cd74	CD74 molecule
10.822	LOC110351111	uncharacterized LOC110351111
11.183	Il21r	interleukin 21 receptor
13.055	Fdcsp	follicular dendritic cell secreted protein
13.151	LOC101704667	"HLA class II histocompatibility antigen, DQ alpha 1 chain"
13.646	Selp	selectin P
13.948	Il1b	interleukin 1 beta
15.231	Mmp9	matrix metallopeptidase 9
15.632	LOC110350305	uncharacterized LOC110350305
18.900	Tac1	tachykinin precursor 1
40.882	Mmp3	matrix metallopeptidase 3
55.712	Rflna	refilin A

Supplementary Table 3. Sequences of shRNA oligonucleotide targeting β-catenin. Restriction sites used to ligate to pLKO1 vector are AgeI and EcoRI at 5' and 3' respectively; linker sequence is characterized with 6 nucleotides CTCGAG.

No	shRNA	Sequence 5' - 3'
**1**	βcat #1	CCGG**GTTGTGAACCTGATCAACTAC**CTCGAGGTAGTTGATCAGGTTCACAACTTTTTG
**2**	βcat #2	CCGG**CTTGGCTATTACAACAGACTG**CTCGAGCAGTCTGTTGTAATAGCCAAGTTTTTG
**3**	ApoF	CCGG**GGTCAGTGCTTCACTTAAA**CTCGAGTTTAAGTGAAGCACTGACCTTTTTG
**4**	Non-target shRNA (Sigma)	CCGG**CAACAAGATGAAGAGCACCAA**CTCGAGTTGGTGCTCTTCATCTTGTTGTTTTT

Supplementary Table 4. Sequences of oligonucleotide used for RT-PCR.

Gene	Sense Primer	Antisense Primer	Bp
CTNNB1	TTCTTGGCTATTACAACAGAC	CATCCCTTCCTGCTTAGTCG	249
TUBB	AAGAACAGCAGCTACTTCG	TTCATGTTGCTCTCGGCCTC	242
CCND1	GTGCATCTACACTGACAACTC	GATCTGTTTGTTCTCCTCAGCC	160
APOF	CCTGTGGATGCCATTCCA	CCTCCAGGGCATTCCTTAGAG	193

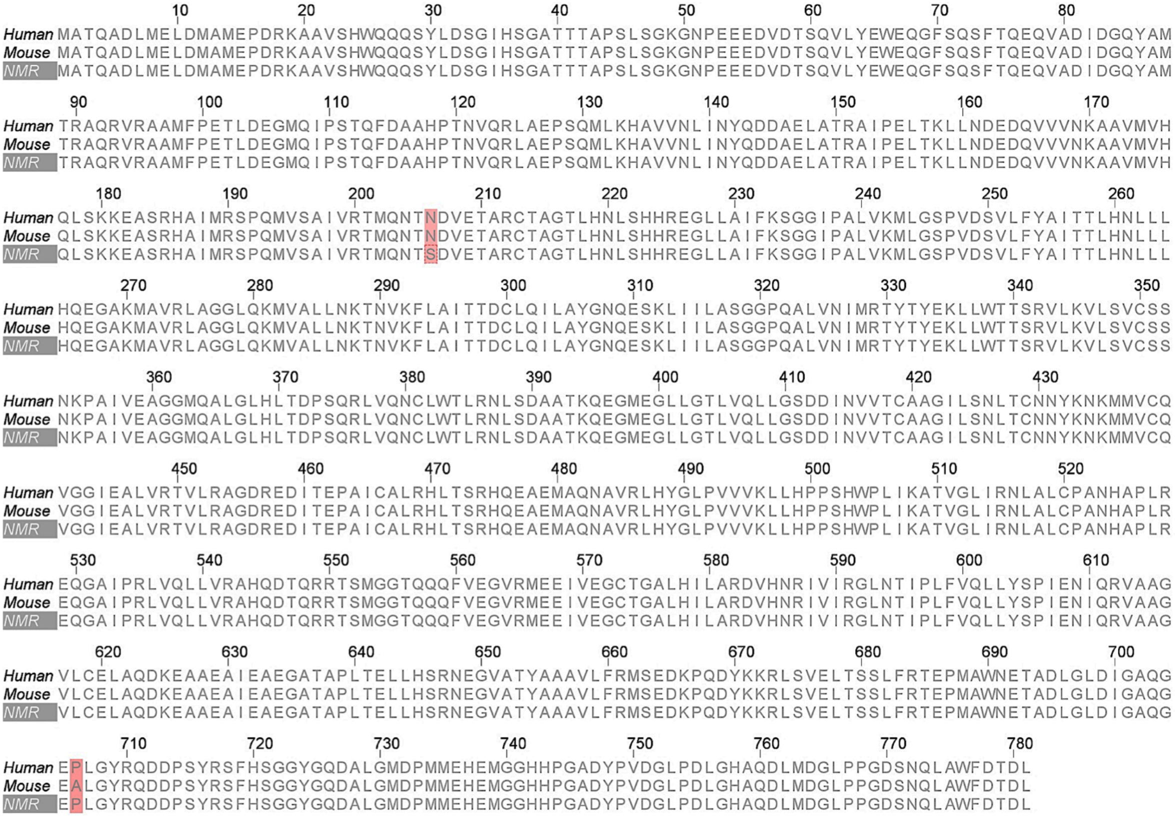

Supplementary Figure 1. Alignment of the human, mouse, and NMR β-catenin amino acid sequences using Clustal W.

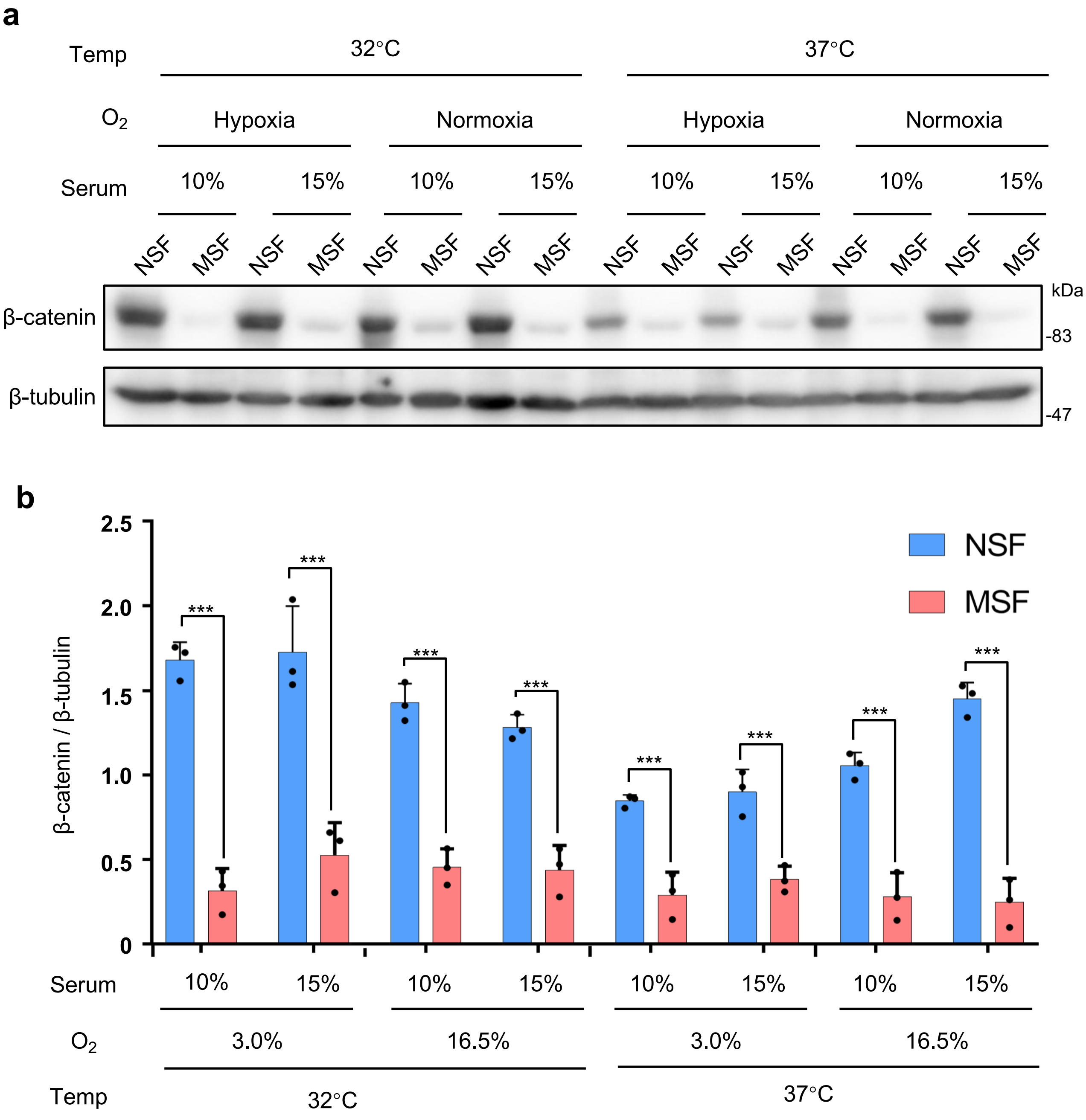

Supplementary Figure 2. Unique supernumerary accumulation of β-catenin in NMR skin fibroblasts (NSFs). (a) Immunoblot showing that β-catenin expression levels were significantly higher in NSFs than in MSFs. β-tubulin was used as a loading control. **(b)** Densitometric quantification of β-catenin/β-tubulin expression from the immunoblot. Data are expressed as the mean ± standard deviation (n=3 biologically independent experiments). ***$p < 0.001$, Student's paired *t*-test.

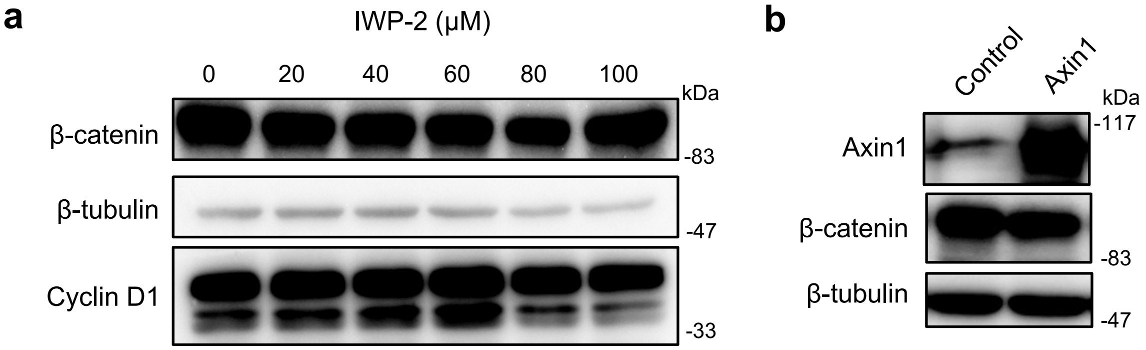

Supplementary Figure 3. Wnt- and axin-independent stabilization of β-catenin accumulation in NSFs. (a) Immunoblot showing β-catenin expression levels in the presence of increasing doses of IWP-2, an inhibitor of Wnt signaling. β-tubulin was used as a loading control. **(b)** Immunoblot showing that overexpression of the scaffold protein *Axin 1* did not affect changes in β-catenin expression levels observed in NMR cells. β-tubulin was used as a loading control.

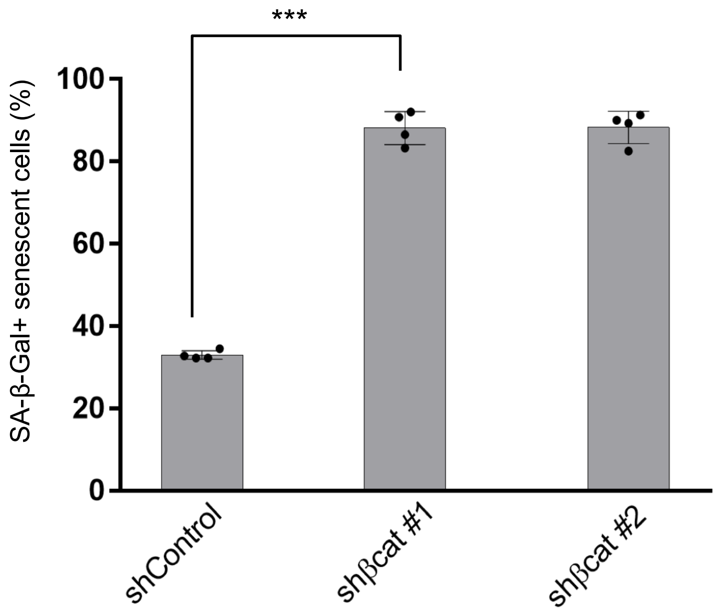

Supplementary Figure 4. β-catenin downregulation induces senescence-like phenotypes in NSFs. Quantitative analysis of SA-β-Gal activity in control and *β-catenin* knockdown NSFs. Data are expressed as the mean ± standard deviation (n=4 biologically independent experiments). ***p<0.001, Student's *t*-test

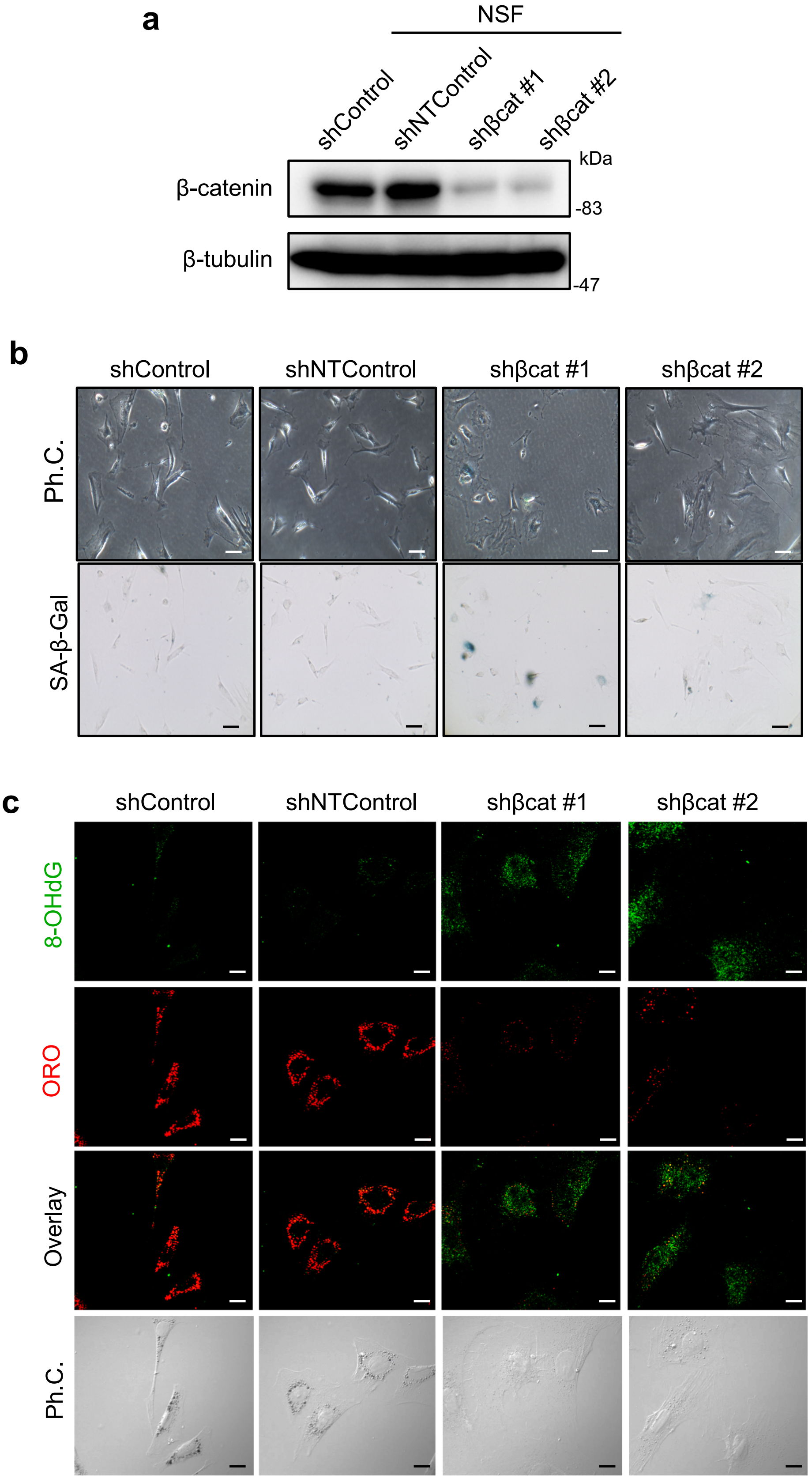

Supplementary Figure 5. Non-targeting knockdown does not change β-catenin expression or lipid droplet abundance. (a) Immunoblot showing β-catenin expression levels upon treatment with Mock (*shControl*), non-target shRNA (*shNTControl*), and *shβ-catenin*. **(b)** Representative phase-contrast and bright-field images showing SA-β-Gal staining in NSFs treated with *shControl*, *shNTcontrol*, or *shβ-catenin*. Scale bars, 100 μm. **(c)** Immunofluorescence staining of NSFs treated with *shControl*, *shNTcontrol*, and shβ-catenin with 8-OHdG (green) and ORO (red). Scale bars, 20 μm.

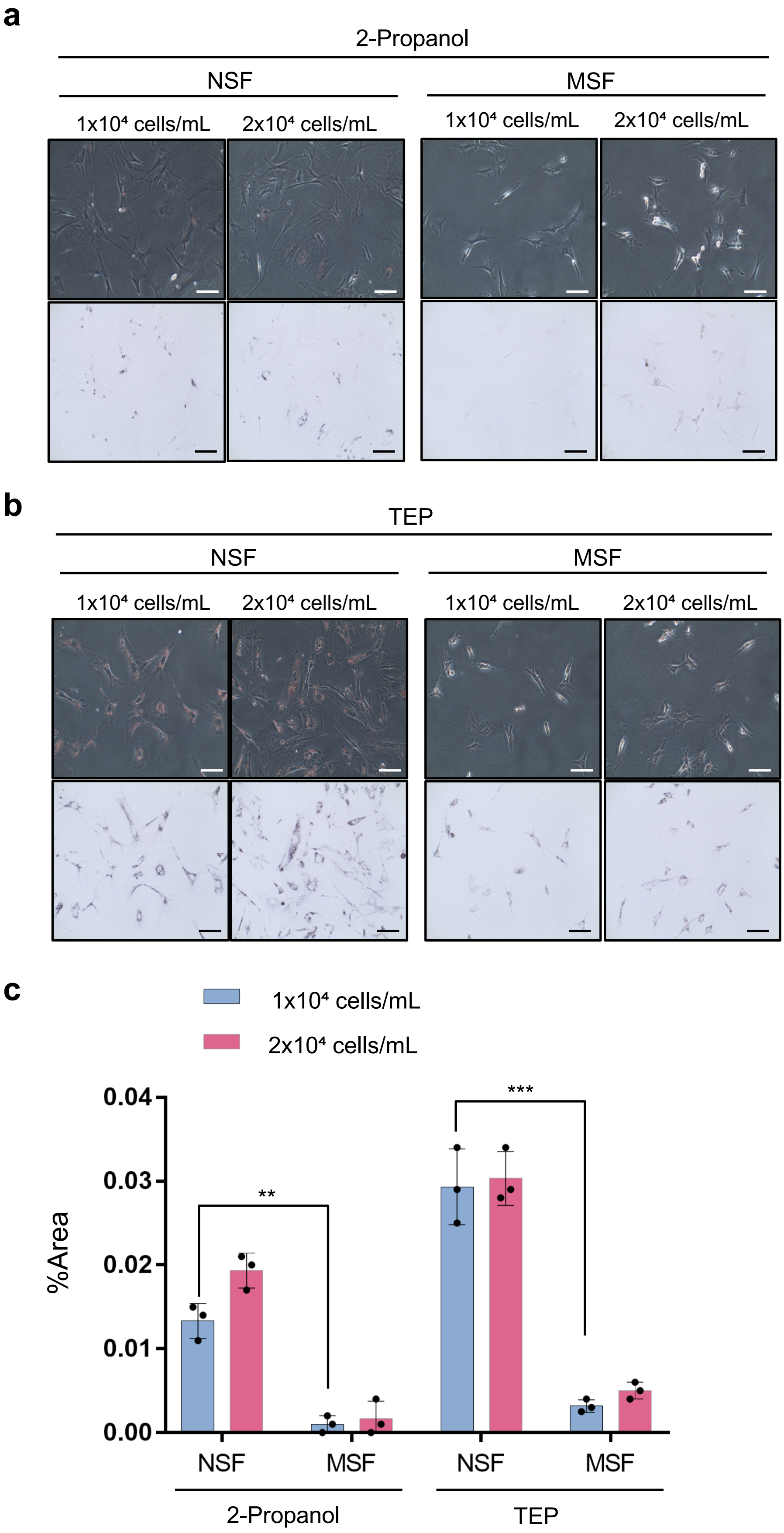

Supplementary Figure 6. (a, b) Representative bright-field images showing lipid droplet staining by ORO in NSFs (a) and MSFs (b) under the indicated conditions. Scale bars, 100 µm. **(c)** Quantitative analysis of ORO-stained NSFs and MSFs under the indicated conditions. The ratio of the stained area to the total area was analysed with Image J software. Data are expressed as the mean ± standard deviation (n=3 biologically independent experiments). **p<0.01 and ***p<0.001, Student's paired *t*-test.

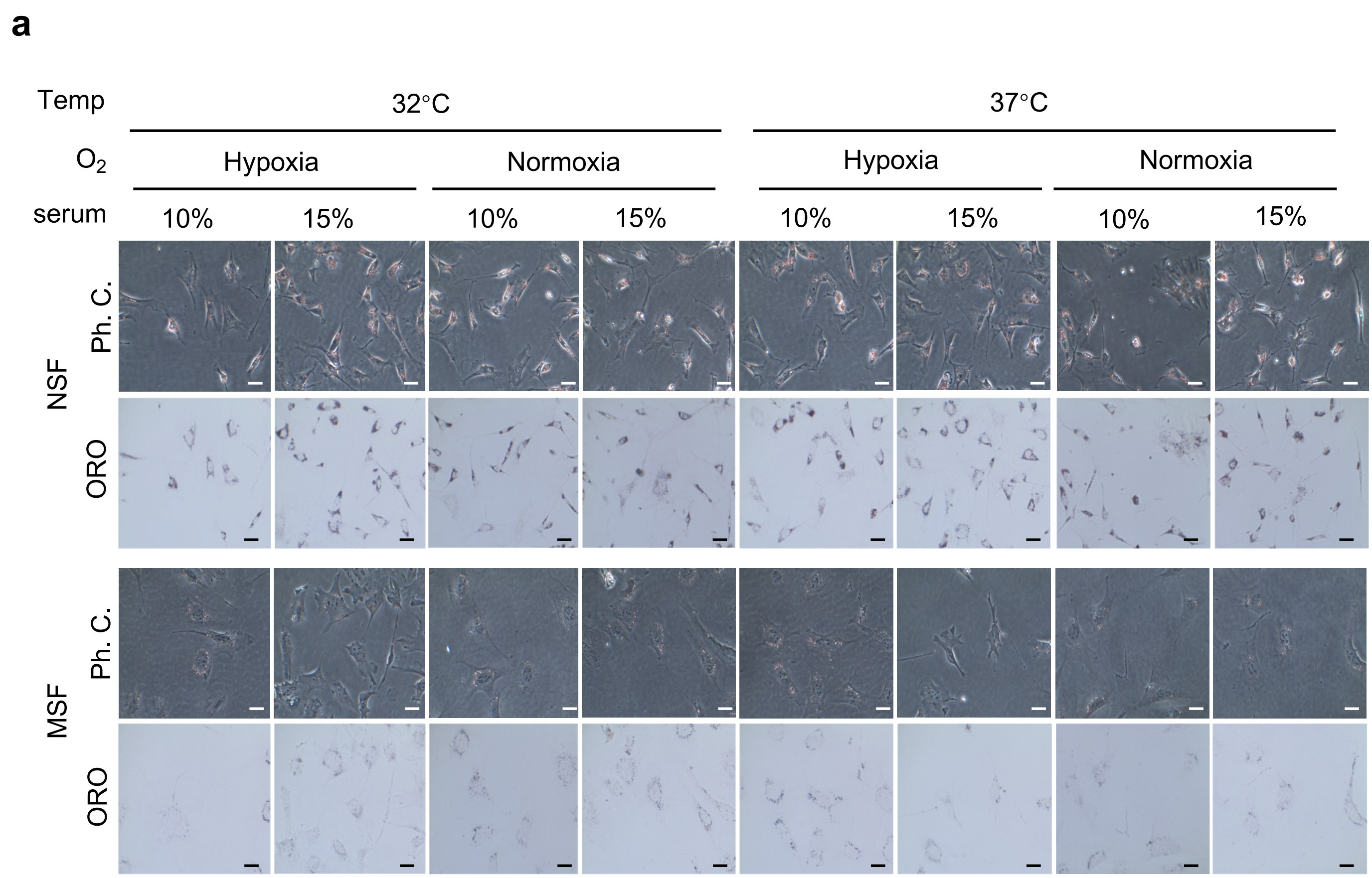

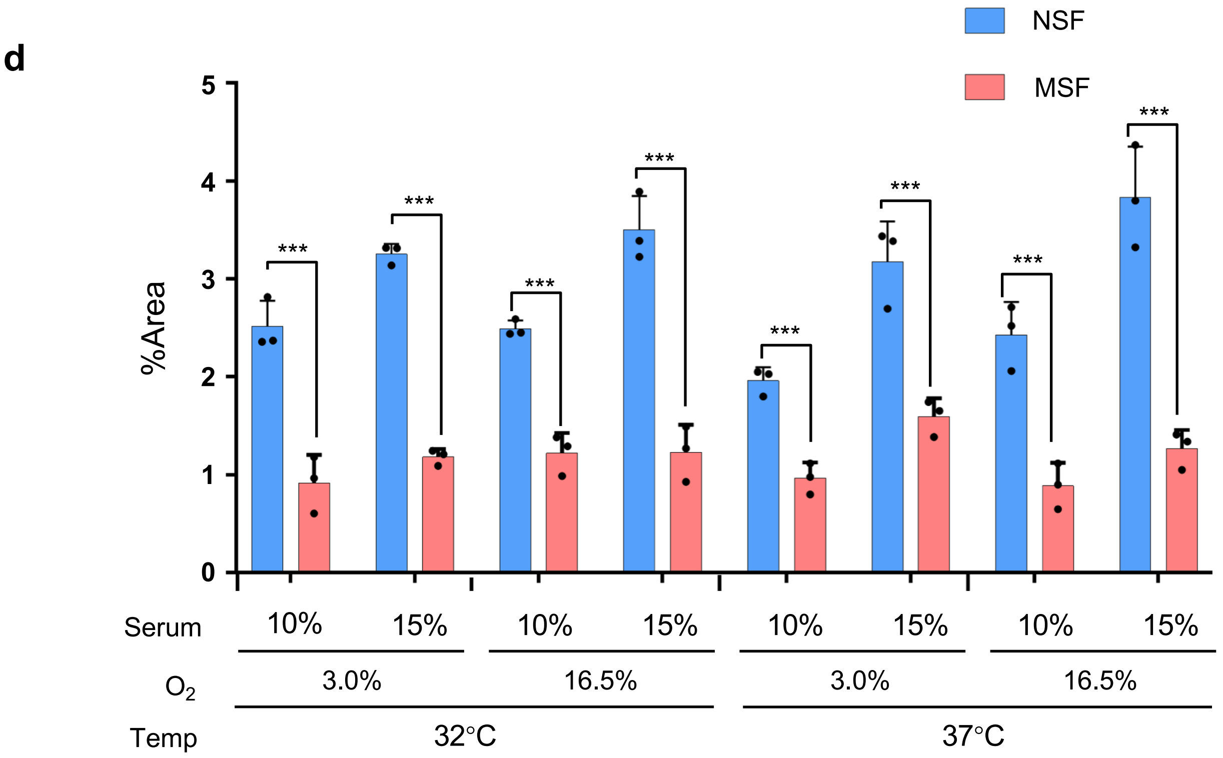

Supplementary Figure 7. Lipid droplets accumulate in NSFs, regardless of culture conditions. **(a)** Representative images showing that stained lipid droplets were significantly more abundant in NSFs than in MSFs under different culture conditions. **(b)** Quantitative results (obtained using ImageJ software) showing percentage coverage of cells stained with ORO over that by total cells. Scale bars, 100 µm. Data are expressed as the mean ± standard deviation (n=3 biologically independent experiments). ***p<0.001, Student's *t*-test.

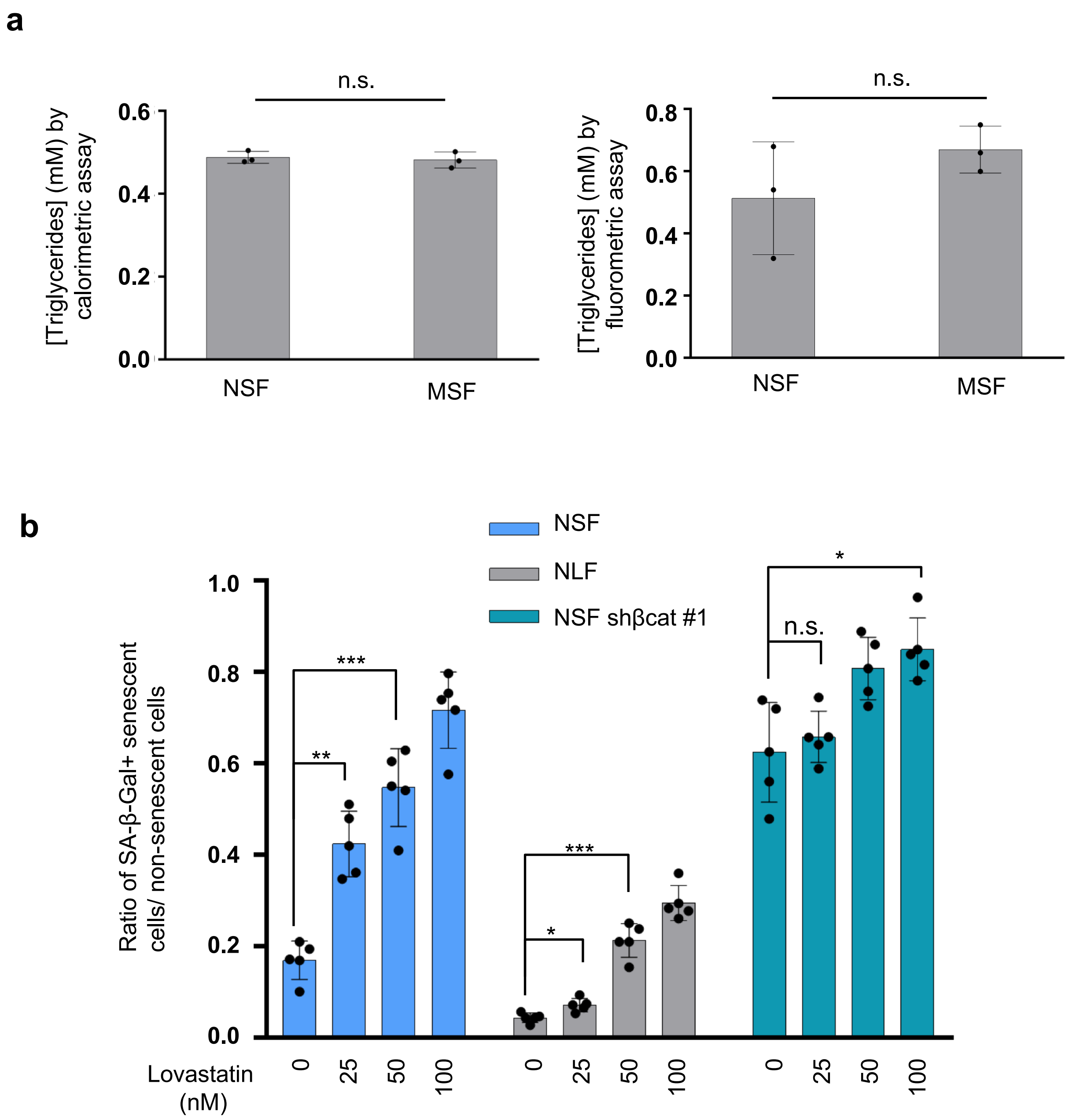

Supplementary Figure 8. NMR cells accumulate lipid droplets consisting primarily of cholesterol. (a) Colorimetric (left) and fluorometric (right) assays comparing triglyceride concentrations between NSFs and MSFs. Data are expressed as the mean ± standard deviation (n=3 biologically independent experiments). n.s, non-significant, Student's unpaired *t*-test . **(b)** Quantitative analysis of SA-β-Gal-stained cells in control NSFs and NLFs, and *β-catenin* knockdown NSFs, which were treated with the indicated concentrations of lovastatin. Data are expressed as the mean ± standard deviation (n=5 biologically independent experiments). n.s, non-significant; *P<0.05, **P<0.01, and ***P<0.001, Student's unpaired *t*-test.

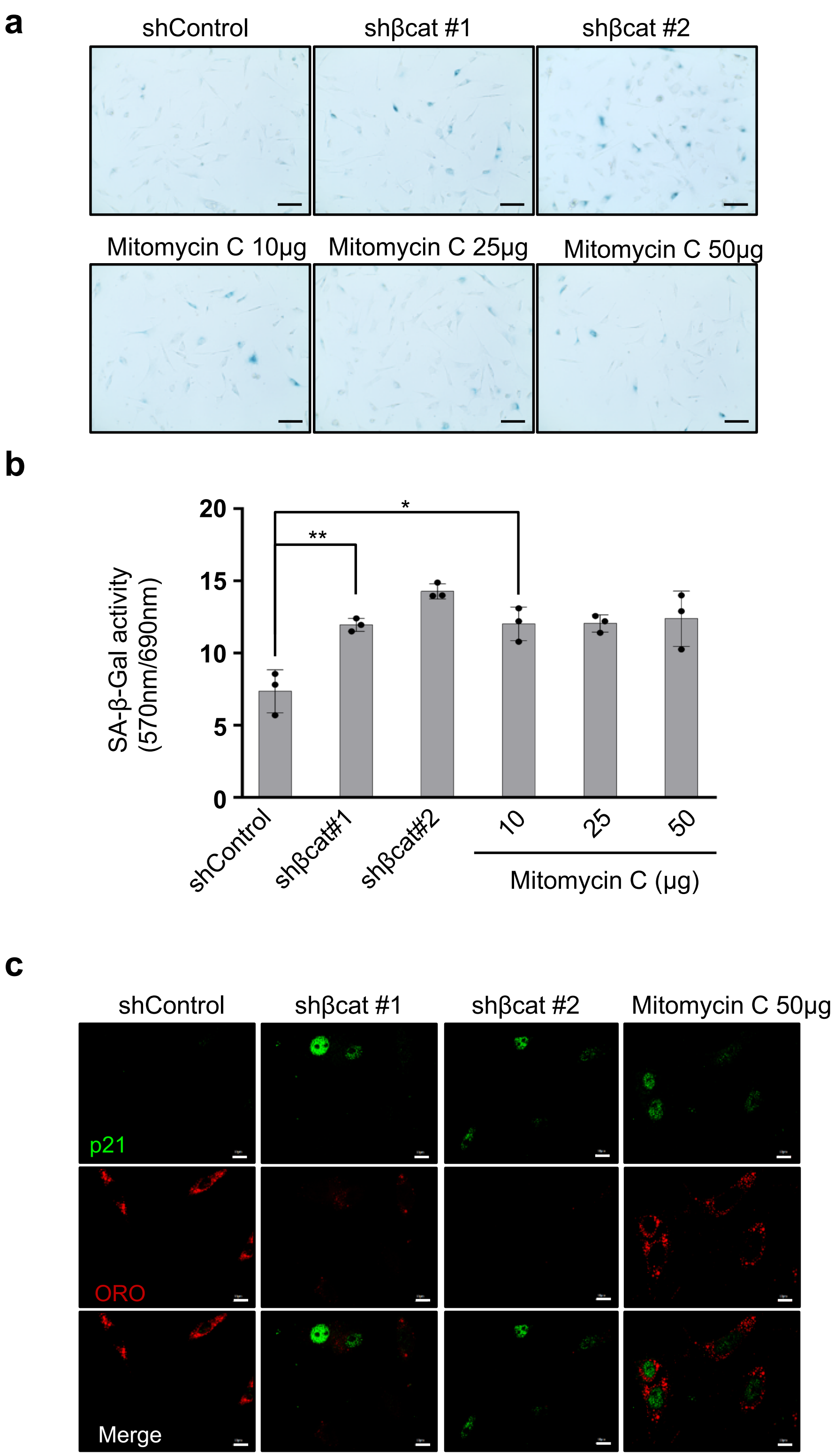

Supplementary Figure 9. NSF lipid droplet formation was not affected by therapy-induced senescence. (a) SA-β-Gal assay in NSFs demonstrating induction of senescence by Mitomycin C. Scale bar, 100 μm. **(b)** CPRG assay quantifying SA-β-Gal activity in NSFs upon Mitomycin C treatment. Data are expressed as the mean ± standard deviation (n=3 biologically independent experiments). * P<0.05 and **p<0.01, Student's unpaired *t*-test. **(c)** Expression of p21 (green) and staining of lipid droplets (red) were observed by confocal microscopy of NSFs subjected to Mitomycin C treatment. Scale bars, 10 μm.

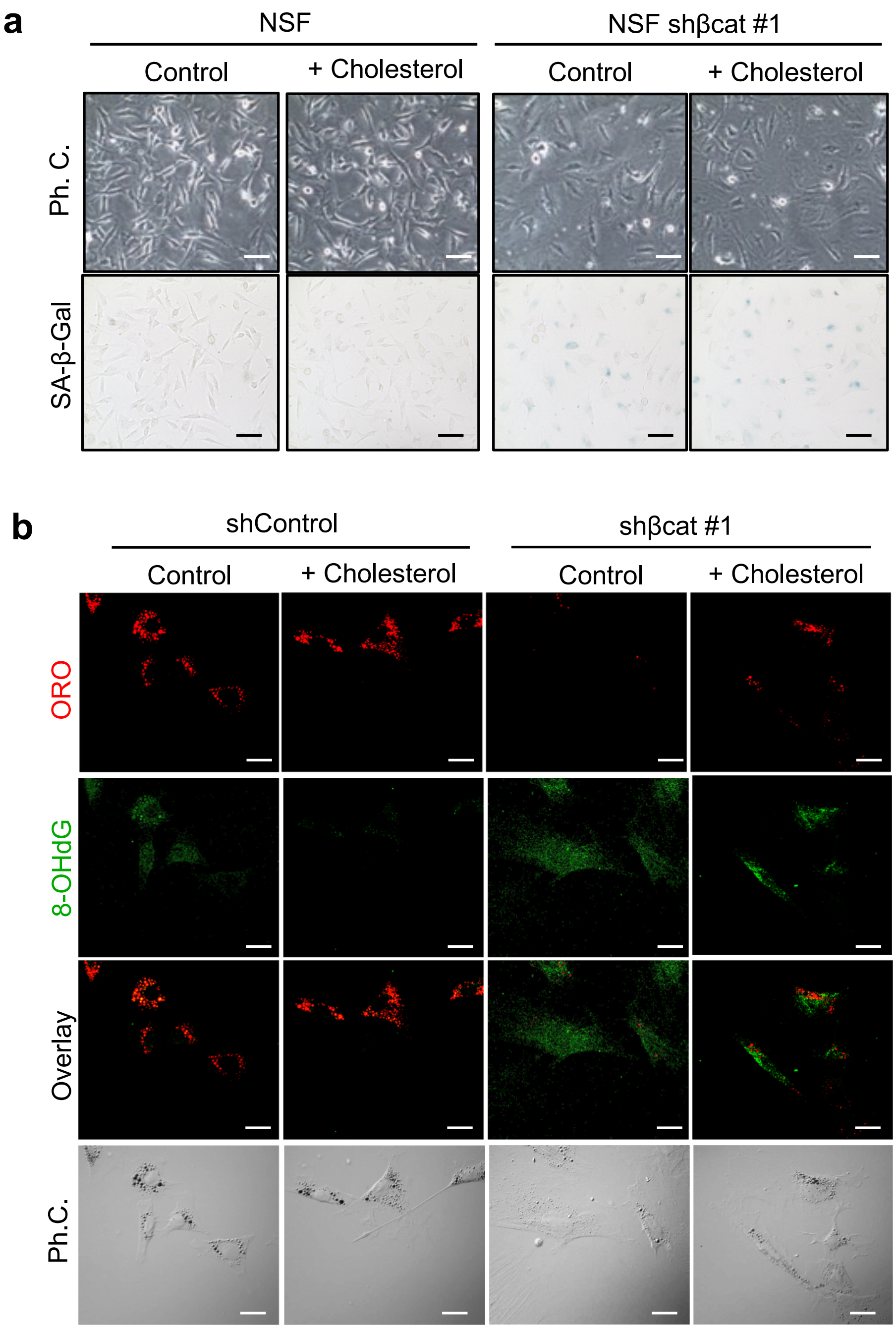

Supplementary Figure 10. Cholesterol loading fails to protect NSFs from cellular senescence under *β-catenin* knockdown conditions. (a) Representative images of SA-β-Gal activity in control NSFs and *β-catenin* knockdown NSFs with or without cholesterol loading. Scale bars, 100 μm. **(b)** Immunofluorescence staining of 8-OHdG (green) and ORO (red) in NSFs and β-catenin knockdown NSFs with or without cholesterol loading (10 μg/mL). Scale bars, 20 μm.

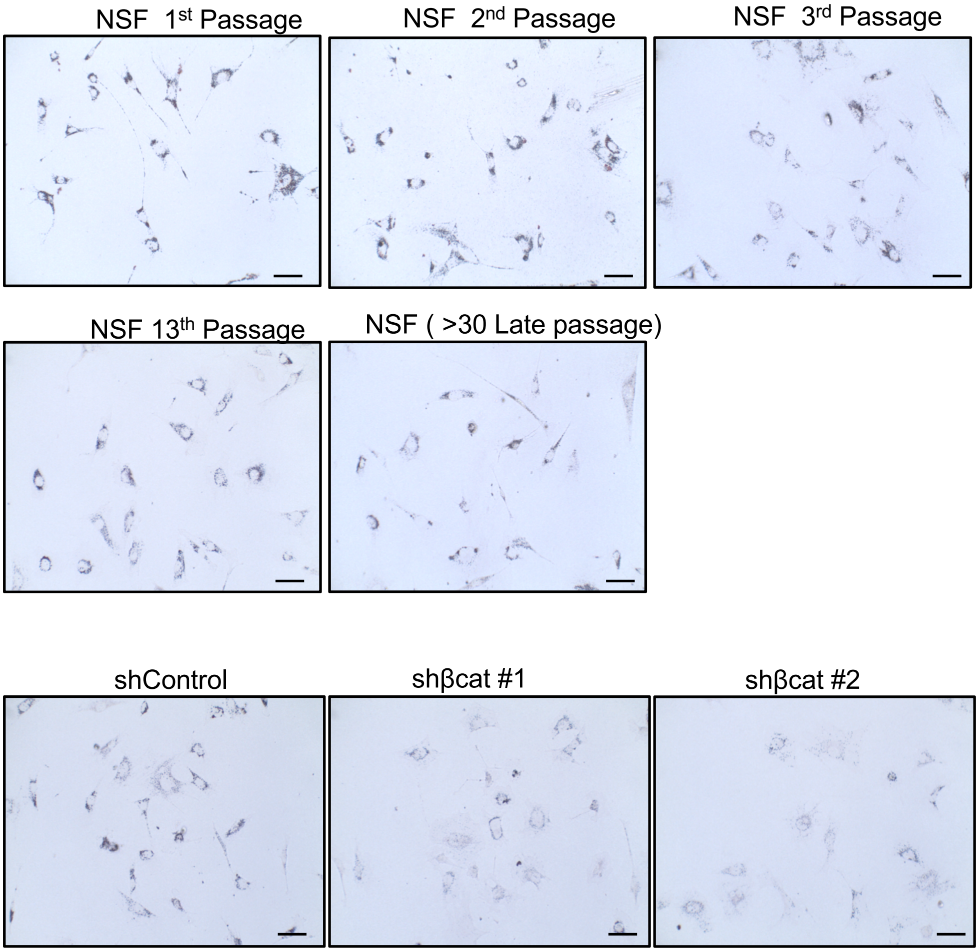

Supplementary Figure 11. Lipid droplet abundance in NSFs is unaffected by passage number. Representative bright-field images showing staining of lipid droplets by ORO in control NSFs at different passages and *β-catenin* knockdown NSFs as a negative control . Scale bars, 100 μm.

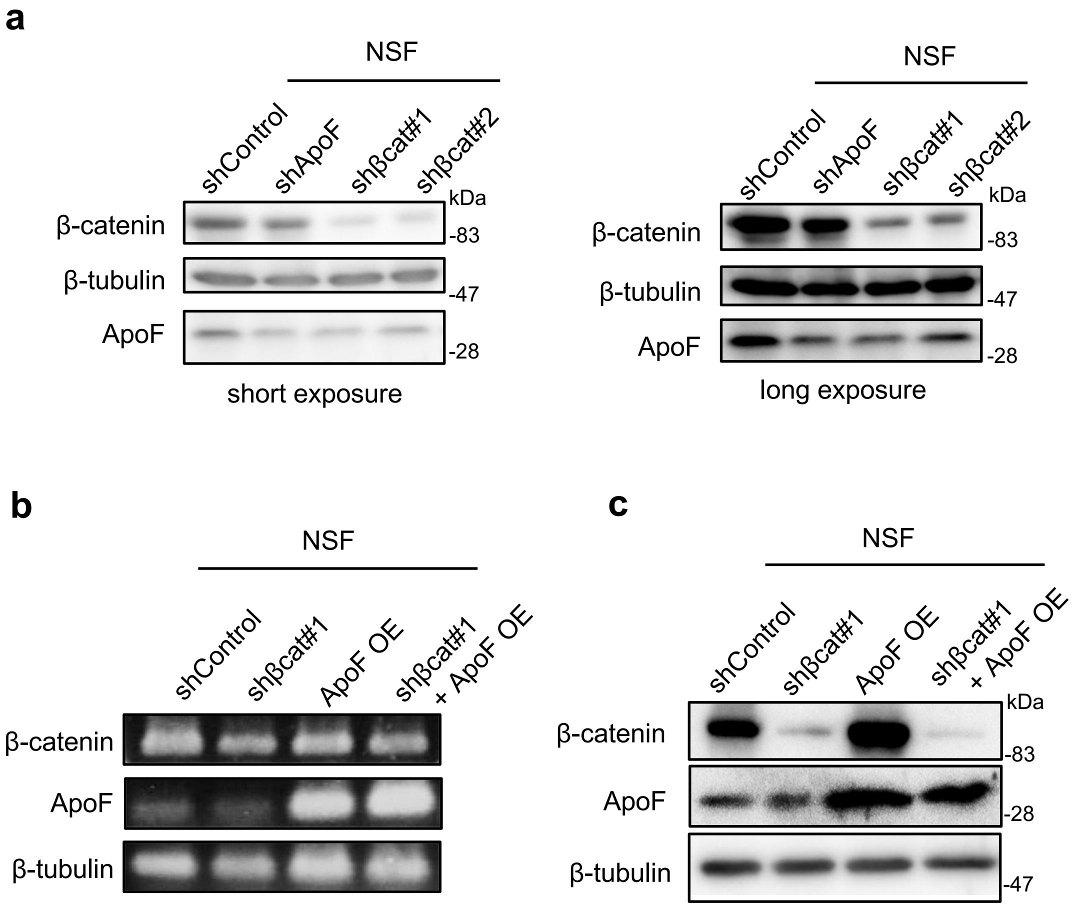

Supplementary Figure 12. ApoF expression is regulated by β-catenin abundance in NSFs. (a) Immunoblot verifying ApoF downregulation upon *β-catenin* or *ApoF* knockdown. **(b)** RT-PCR quantifying expression of *β-catenin* and *ApoF*. (c) Immunoblot showing expression of β-catenin and ApoF upon *β-catenin* knockdown and/or *ApoF* overexpression.

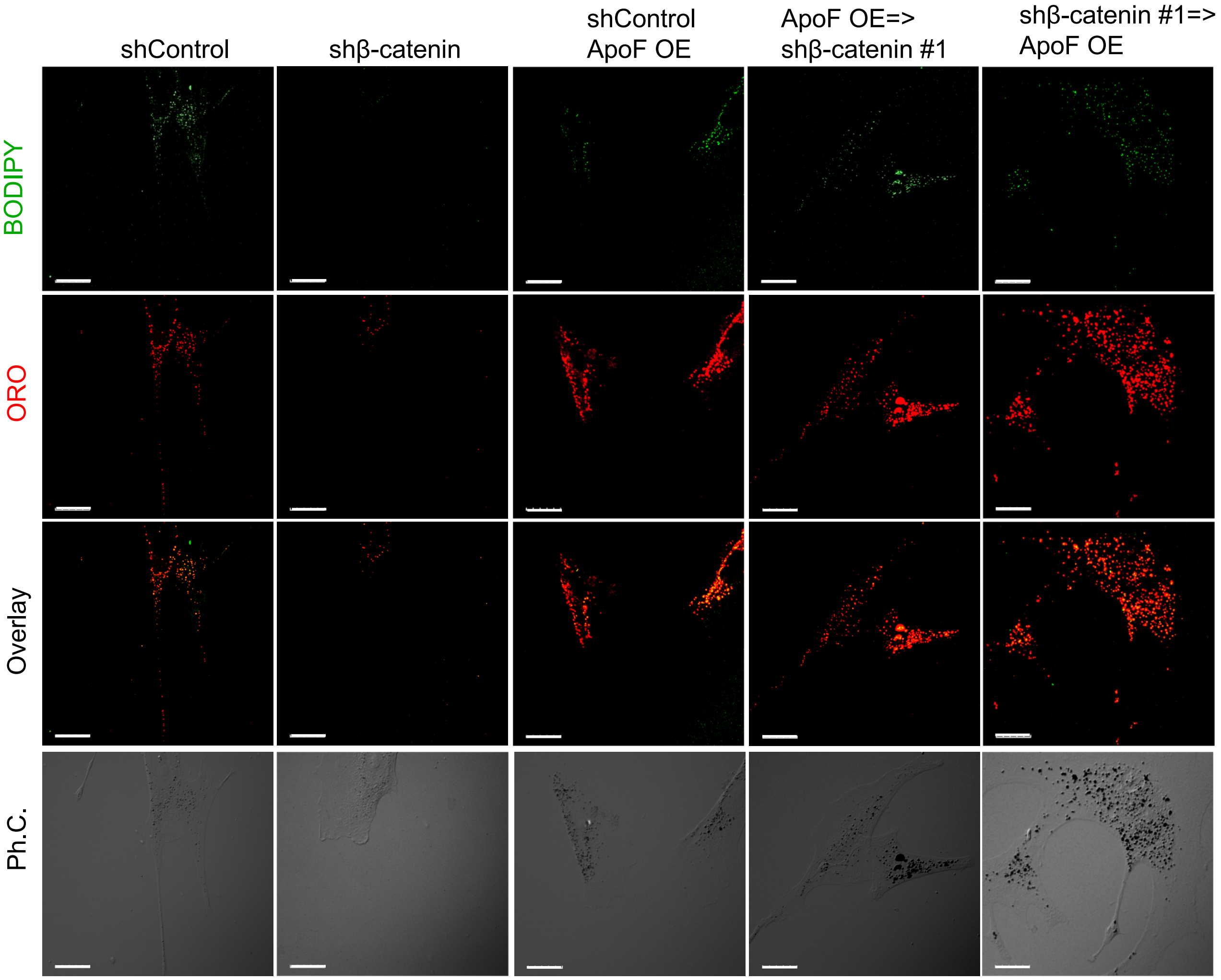

Supplementary Figure 13. Overexpression of ApoF restores formation of cholesterol-enriched lipid droplets, which were suppressed by β-catenin knockdown. Immunofluorescence images demonstrating changes in formation of BODIPY- and ORO-positive lipid droplets upon β-catenin knockdown and/or ApoF overexpression. Scale bars, 20 μm.

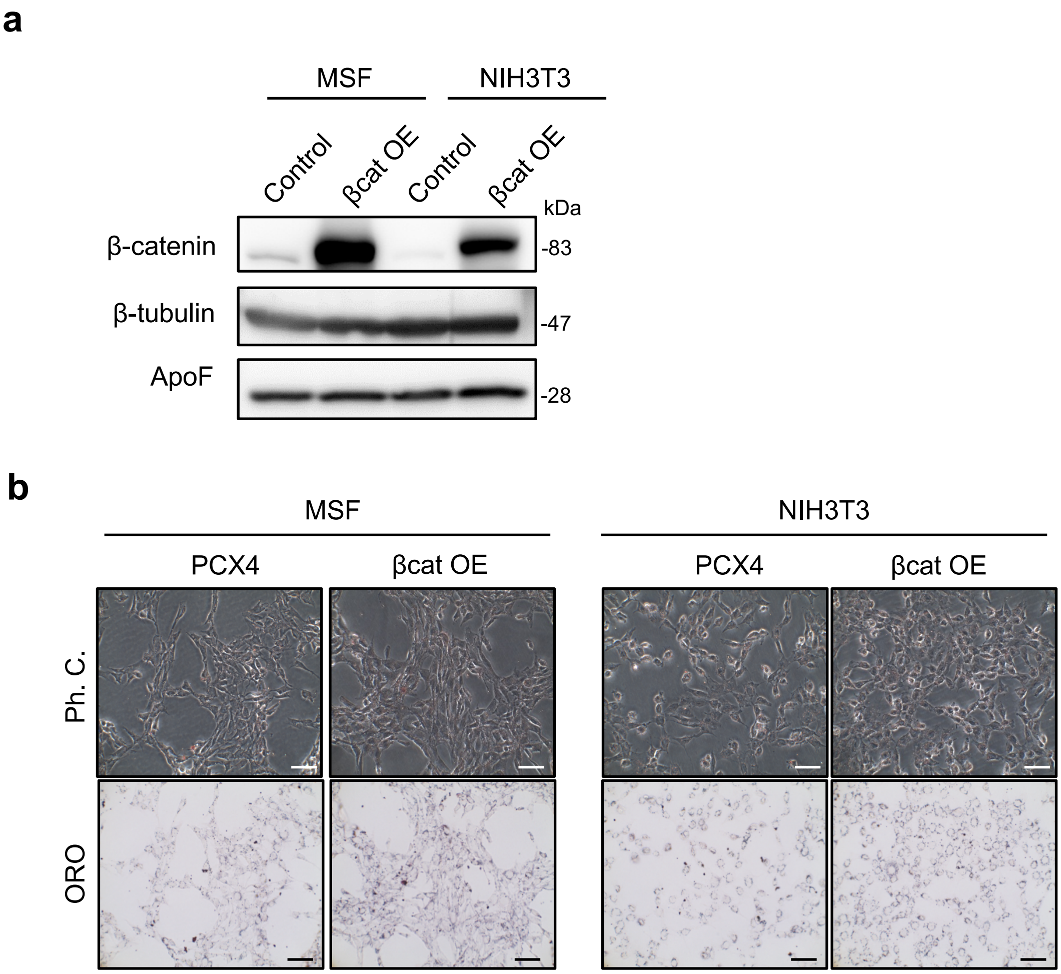

Supplementary Figure 14. Overexpression of β-catenin in MSFs does not alter ApoF expression or lipid droplet abundance. (a) Immunoblot showing expression of β-catenin and ApoF in mouse cell lines after ectopic expression of *β-catenin*. **(b)** Representative phase-contrast and bright-field images of ORO-stained lipid droplets in control and *β-catenin*-overexpressing mouse cell lines. Scale bars, 100 µm.

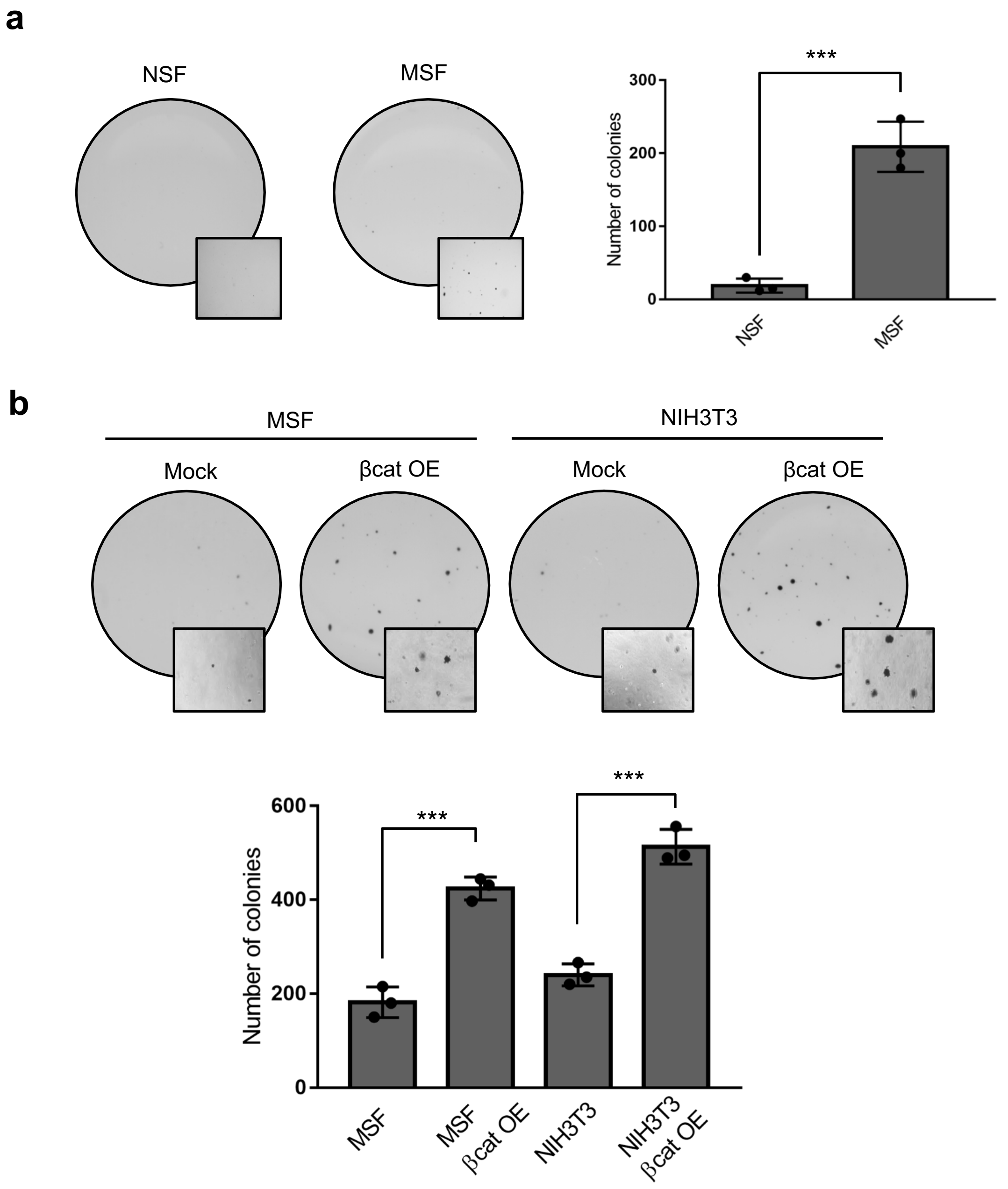

Supplementary Figure 15. Differential role of β-catenin in NMR and mouse cells. **(a)** Soft agar colony formation assay of NSFs and MSFs. Representative dishes and enlarged views are shown (left panels). **(b)** Soft agar colony formation assay for MSFs and NIH3T3 cells with or without overexpression of *β-catenin*. Representative dishes and enlarged views are shown (upper panels). Data are expressed as the mean ± standard deviation (n=3 biologically independent experiments). ***p<0.001, Student's unpaired *t*-test.

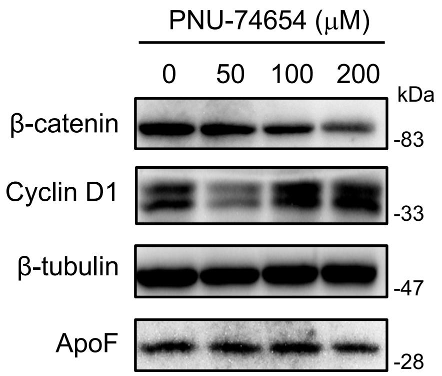

Supplementary Figure 16. ApoF expression is TCF-independent. Immunoblot showing β-catenin expression levels upon treatment with increasing doses of Wnt/β-catenin antagonist PNU-74654.

Original immunoblots in Figure 1a.

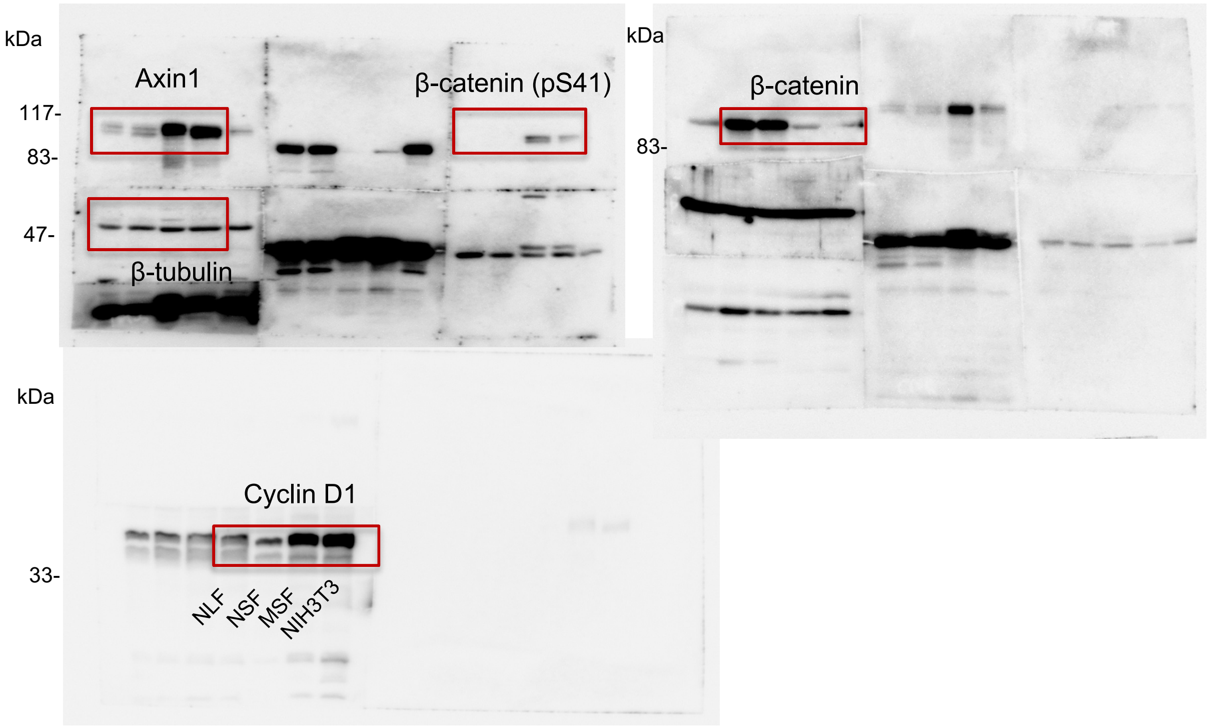

Original immunoblots in Figure 2a.

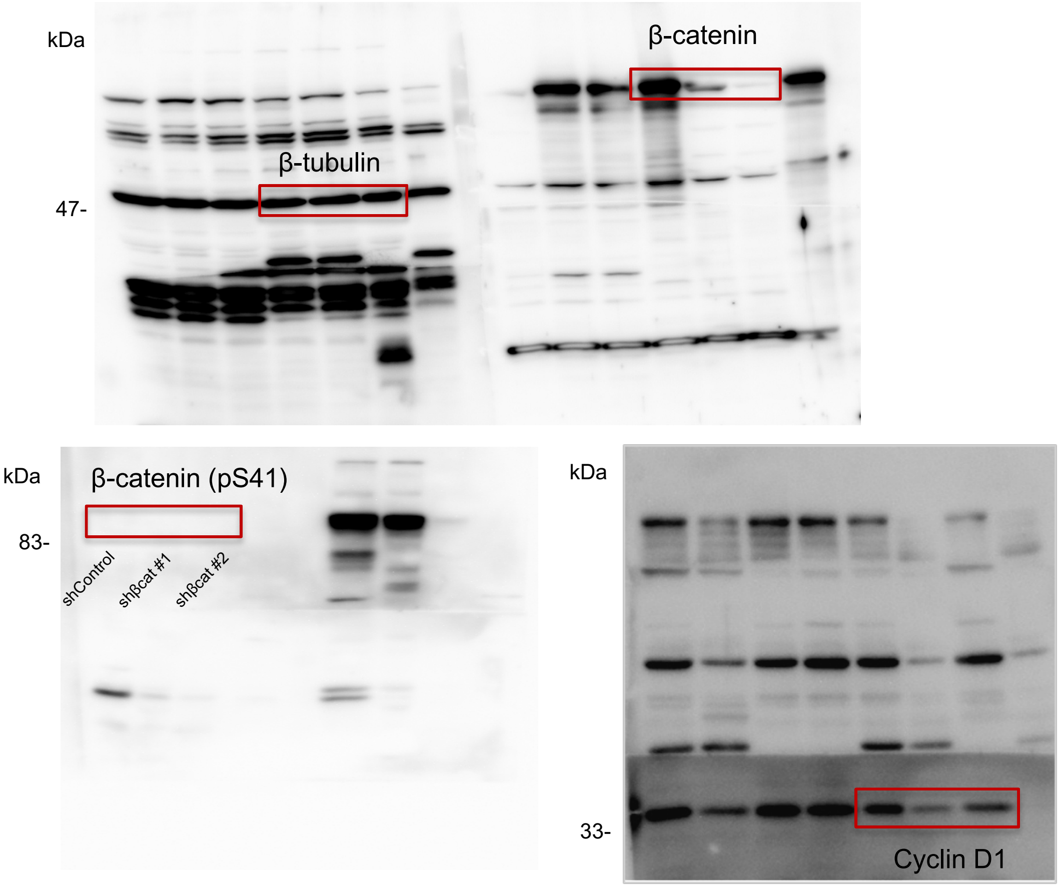

Original gel image in Figure 5c

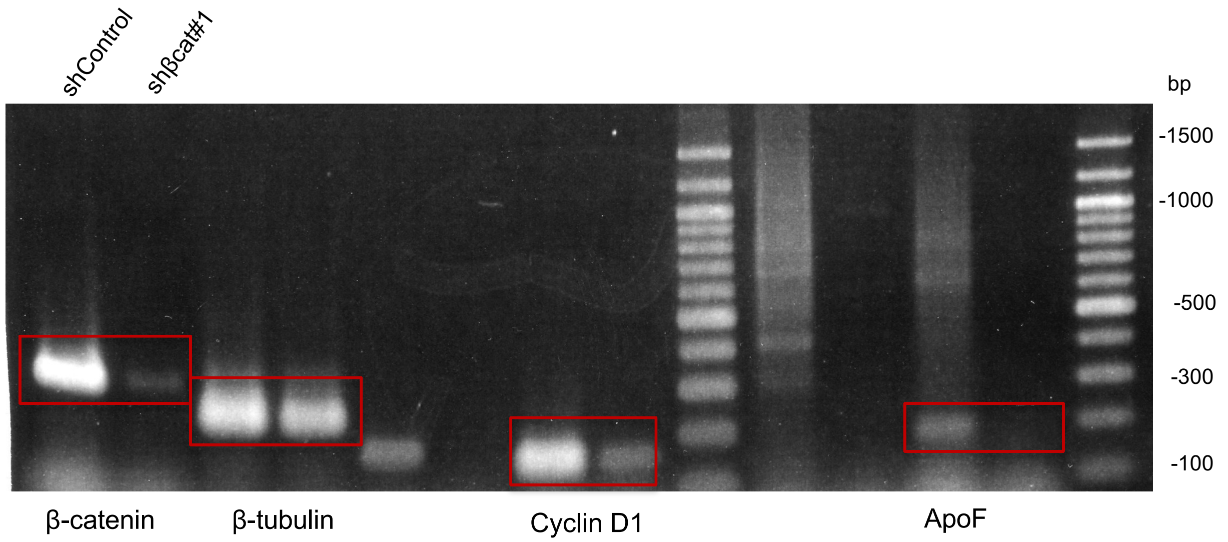

Original immunoblots in Figure 6a

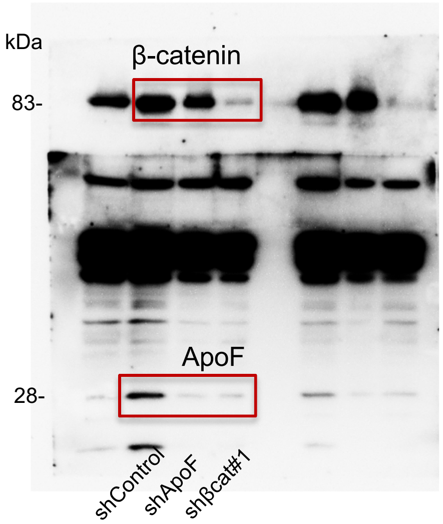

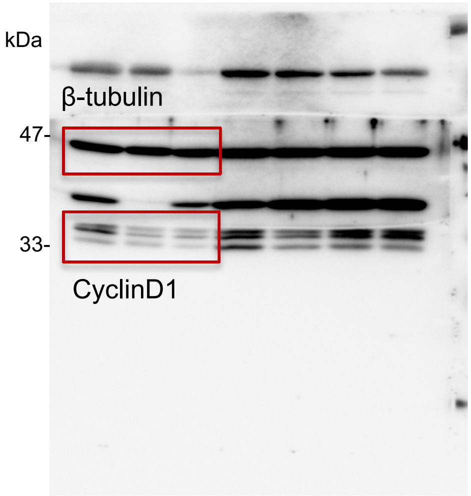

Original immunoblots in Supplementary Figure 2a.

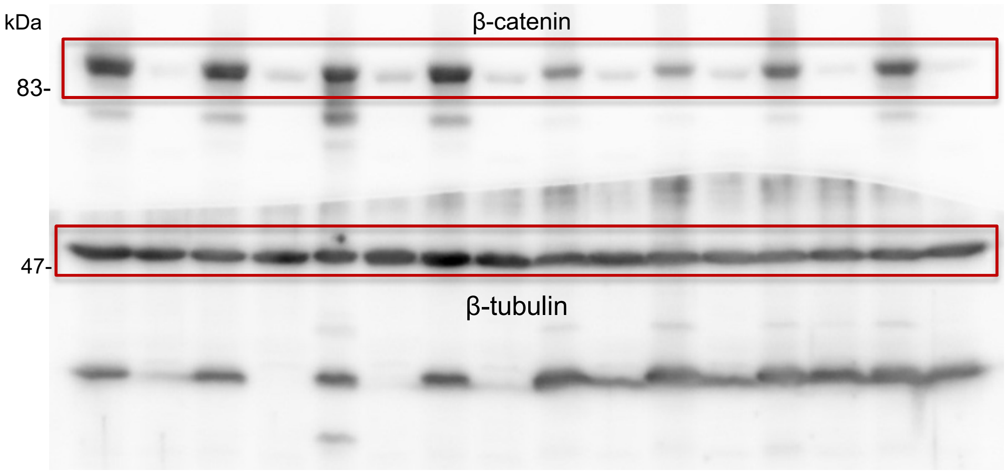

Original immunoblots in Supplementary Figure 3a.

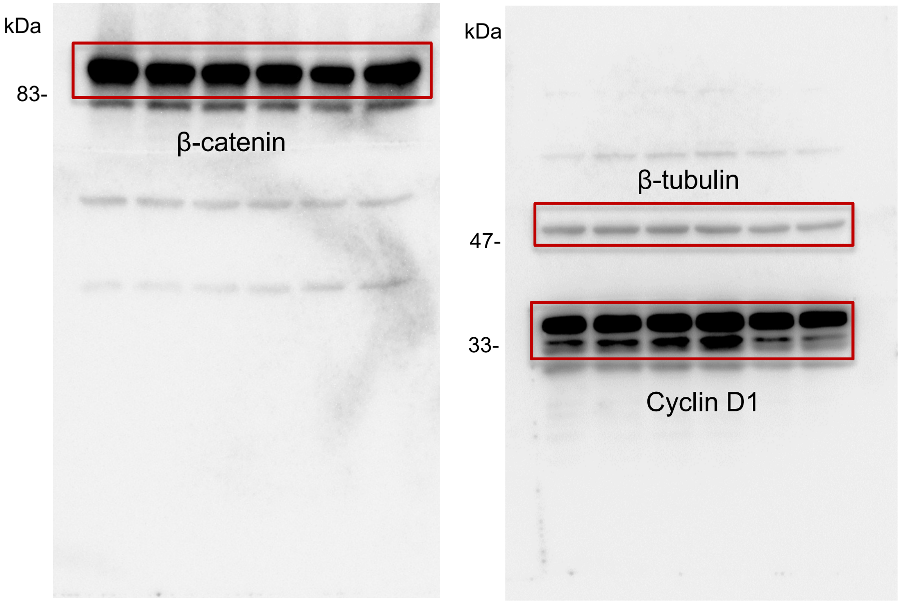

Original immunoblots in Supplementary Figure 3b.

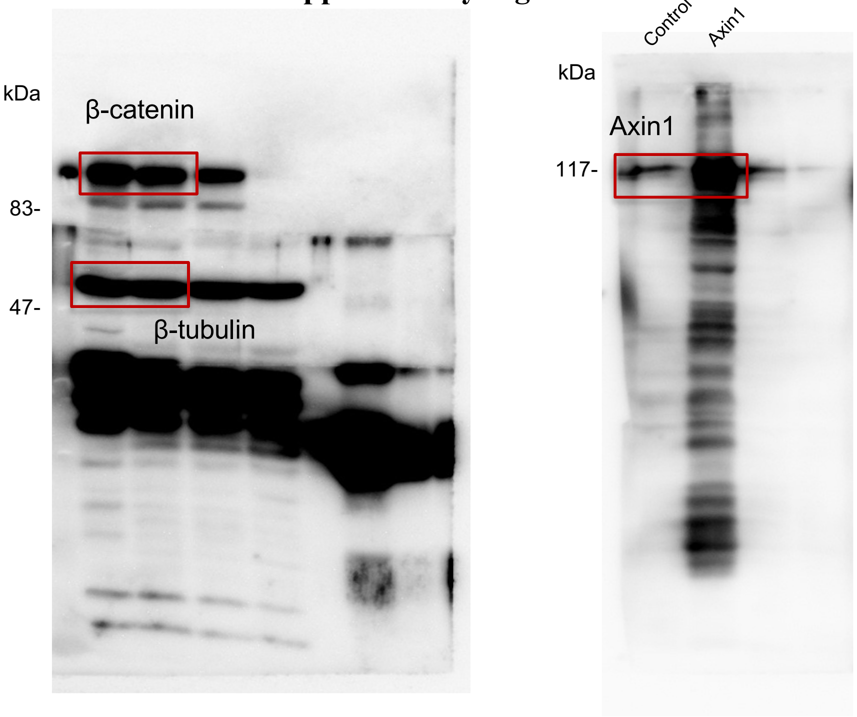

Original immunoblots in Supplementary Figure 5a.

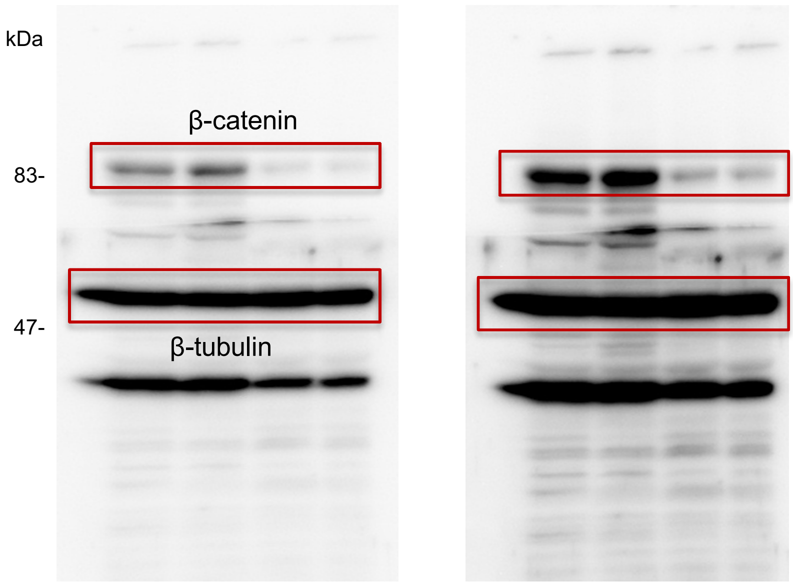

Original immunoblots in Supplementary Figure 12a.

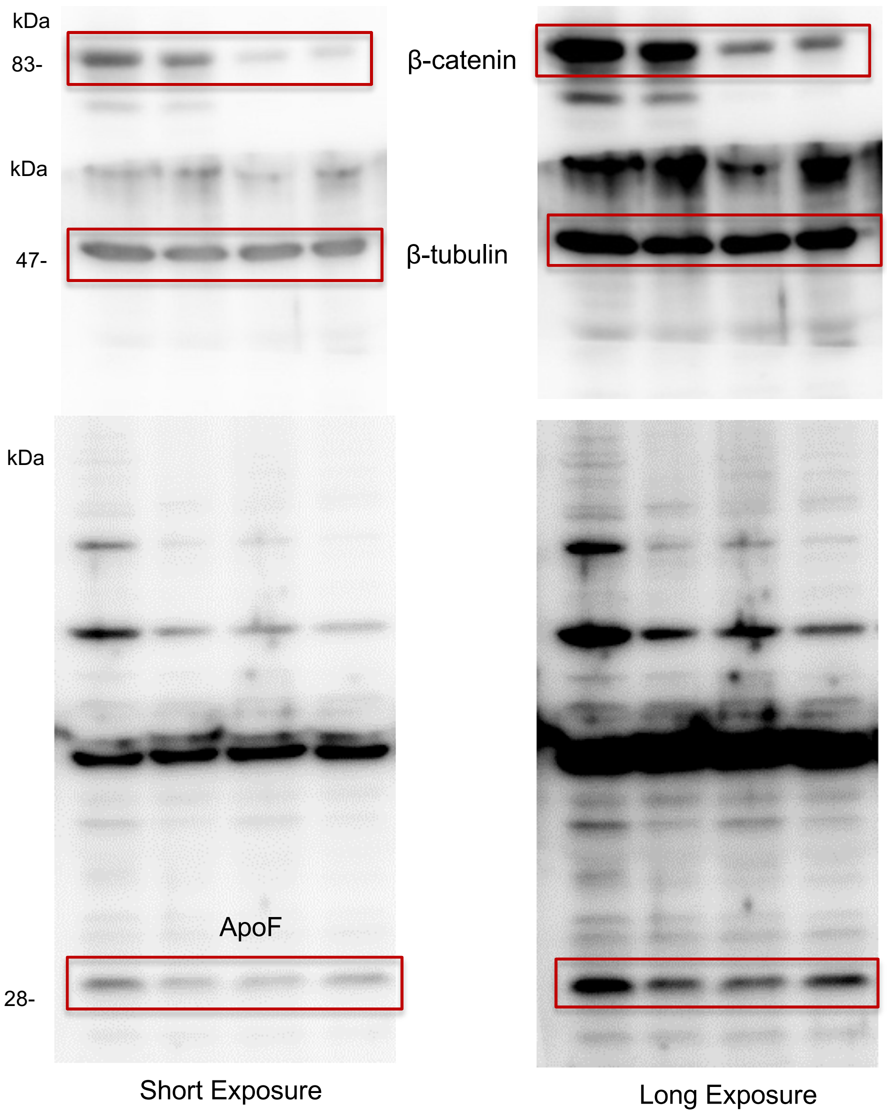

Original gel image in Supplementary Figure 12b.

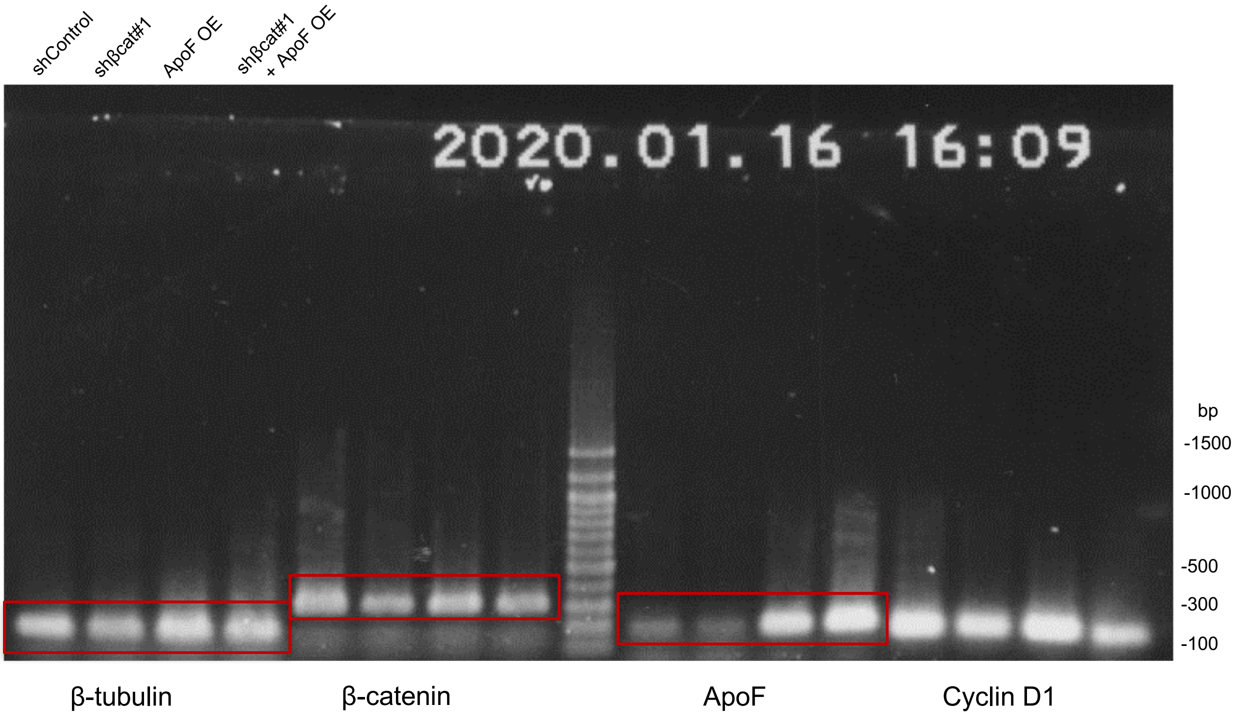

Original immunoblots in Supplementary Figure 12c.

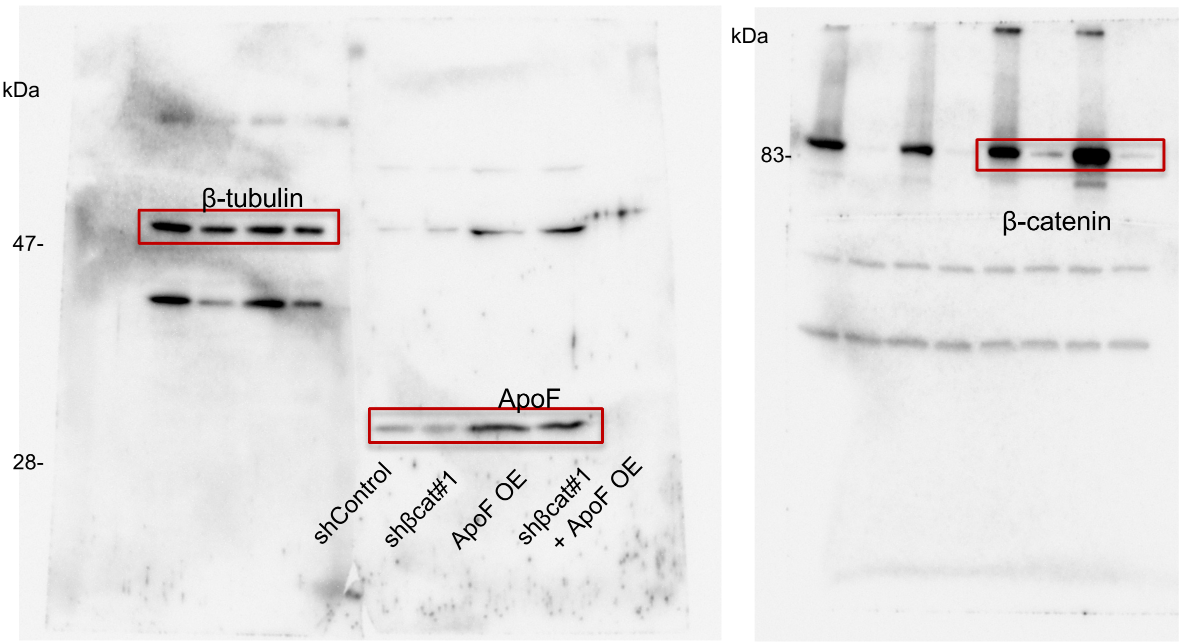

Original immunoblots in Supplementary Figure 14a.

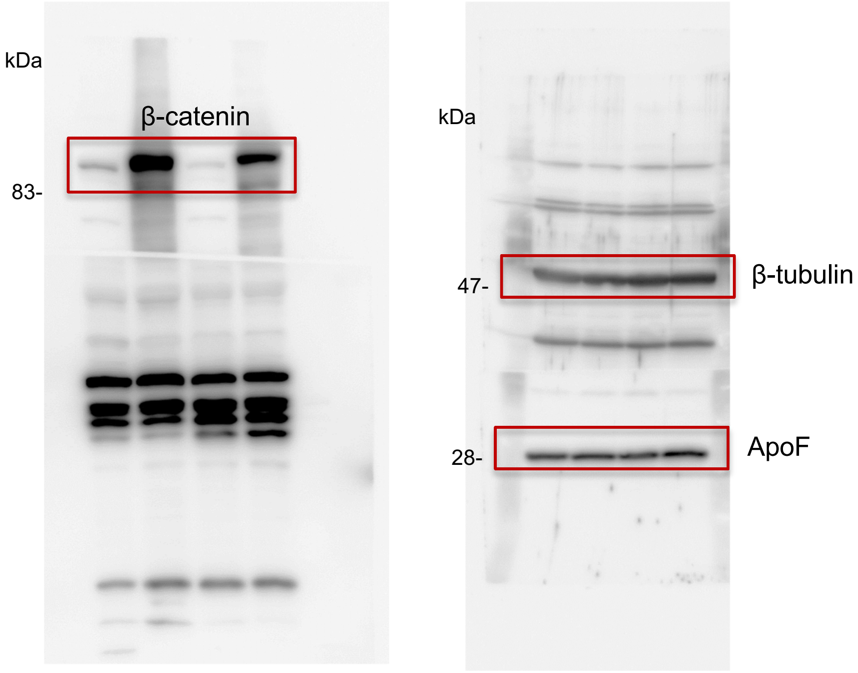

Original immunoblots in Supplementary Figure 16.

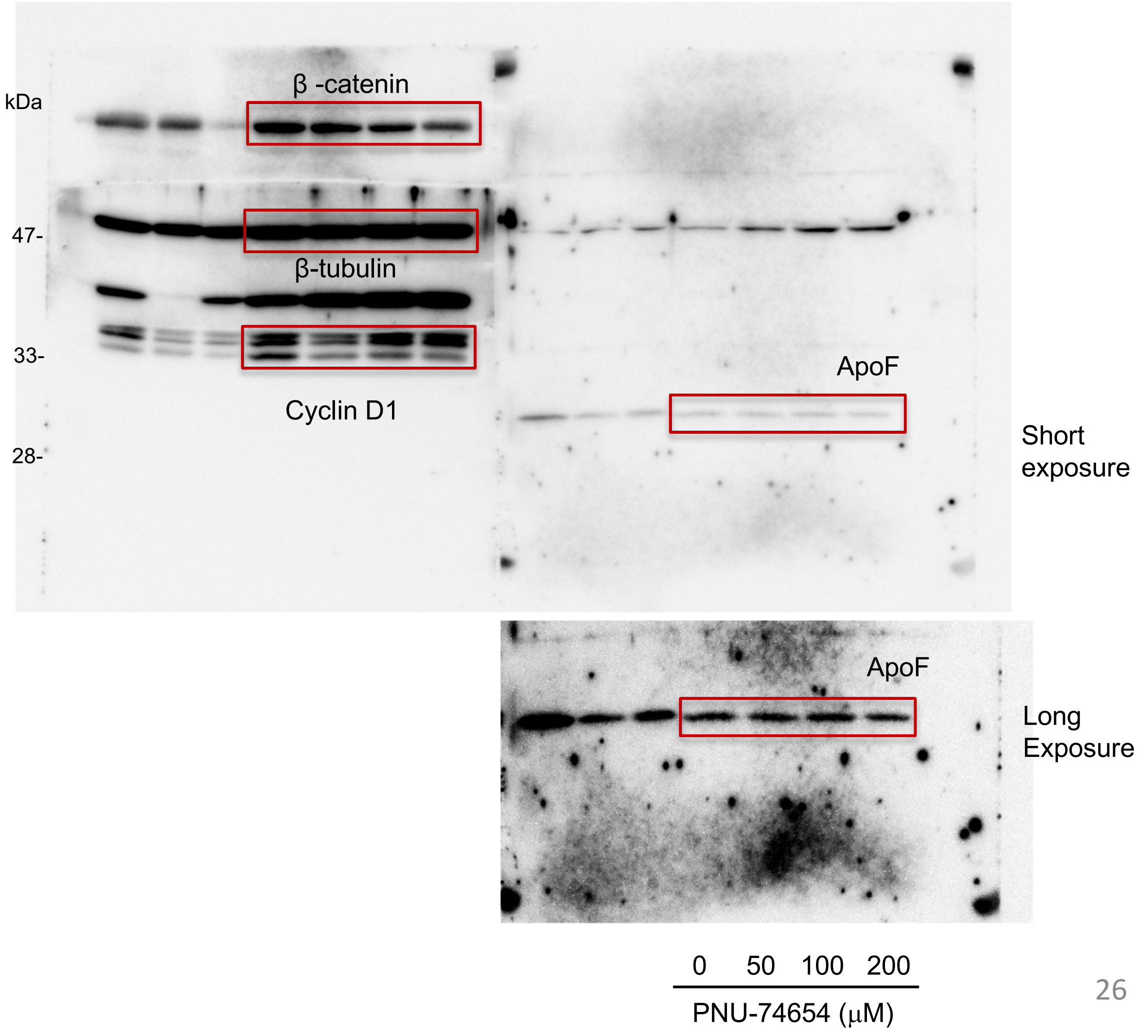

Supplement: Supplementary file 2 — Supplementary information [file 42003_2021_1879_MOESM2_ESM.pdf]
