# Supplementary material for: β-catenin-promoted cholesterol metabolism protects against cellular senescence in naked mole-rat cells
Source: Commun Biol. 2021 Mar 19;4:357. doi: 10.1038/s42003-021-01879-8 (PMC7979689; doi:10.1038/s42003-021-01879-8)
Supplement: Supplementary file 3 — Description of Additional Supplementary Files [file 42003_2021_1879_MOESM3_ESM.pdf]

Description of Additional Supplementary Files

File Name: Supplementary Data 1

Description: Source data underlying the graphs in the figures (Chee et al).
