# Supplementary material for: β-catenin-promoted cholesterol metabolism protects against cellular senescence in naked mole-rat cells
Source: Commun Biol. 2021 Mar 19;4:357. doi: 10.1038/s42003-021-01879-8 (PMC7979689; doi:10.1038/s42003-021-01879-8)
Supplement: Supplementary file 5 — Reporting Summary [file 42003_2021_1879_MOESM5_ESM.pdf]

nature research

Corresponding author(s): Masato Okada

Last updated by author(s): Feb 5, 2021

Reporting Summary

Nature Research wishes to improve the reproducibility of the work that we publish. This form provides structure for consistency and transparency in reporting. For further information on Nature Research policies, see our Editorial Policies and the Editorial Policy Checklist.

Statistics

For all statistical analyses, confirm that the following items are present in the figure legend, table legend, main text, or Methods section.

n/a	Confirmed	
☐	☒	The exact sample size (n) for each experimental group/condition, given as a discrete number and unit of measurement
☐	☒	A statement on whether measurements were taken from distinct samples or whether the same sample was measured repeatedly
☐	☒	The statistical test(s) used AND whether they are one- or two-sided<br>*Only common tests should be described solely by name; describe more complex techniques in the Methods section.*
☐	☒	A description of all covariates tested
☐	☒	A description of any assumptions or corrections, such as tests of normality and adjustment for multiple comparisons
☐	☒	A full description of the statistical parameters including central tendency (e.g. means) or other basic estimates (e.g. regression coefficient) AND variation (e.g. standard deviation) or associated estimates of uncertainty (e.g. confidence intervals)
☐	☒	For null hypothesis testing, the test statistic (e.g. F, t, r) with confidence intervals, effect sizes, degrees of freedom and P value noted<br>*Give P values as exact values whenever suitable.*
☒	☐	For Bayesian analysis, information on the choice of priors and Markov chain Monte Carlo settings
☒	☐	For hierarchical and complex designs, identification of the appropriate level for tests and full reporting of outcomes
☒	☐	Estimates of effect sizes (e.g. Cohen's d, Pearson's r), indicating how they were calculated

Our web collection on statistics for biologists contains articles on many of the points above.

Software and code

Policy information about availability of computer code

Data collection	No software was used
Data analysis	Utilization of software for analysis has been included in the Methods. Cells stained with X-Gal or ORO in SA-B-Gal assay and ORO assay were quantified using ImageJ software. Computational analysis for RNA-seq data were performed using Qiagen_IPA software. Quantification of colonies in soft-agar colony formation assay were performed using Metamorph Version 7.8.8.0.

For manuscripts utilizing custom algorithms or software that are central to the research but not yet described in published literature, software must be made available to editors and reviewers. We strongly encourage code deposition in a community repository (e.g. GitHub). See the Nature Research guidelines for submitting code & software for further information.

Data

Policy information about availability of data

All manuscripts must include a data availability statement. This statement should provide the following information, where applicable:
- Accession codes, unique identifiers, or web links for publicly available datasets
- A list of figures that have associated raw data
- A description of any restrictions on data availability

Data available within the article or its supplementary materials. For RNA-seq raw data, the following secure token was created to allow review of records (GSE147871) while retaining private status: svgjmwkaztivdet. To review GEO accession GSE147871, go to https://www.ncbi.nlm.nih.gov/geo/query/acc.cgi?acc=GSE147871 and enter the password svgjmwkaztivdet .

April 2020

Field-specific reporting

Please select the one below that is the best fit for your research. If you are not sure, read the appropriate sections before making your selection.

- [x] Life sciences
- [ ] Behavioural & social sciences
- [ ] Ecological, evolutionary & environmental sciences

For a reference copy of the document with all sections, see nature.com/documents/nr-reporting-summary-flat.pdf

Life sciences study design

All studies must disclose on these points even when the disclosure is negative.

Sample size	The sample size for the number of cells were seeded according to the size of the plate's wells as stated in Methods. For imaging experiments, between three to six images of cellular morphologies and phenotypes were captured at random position per experimental paradigm.
Data exclusions	No data were excluded from this research.
Replication	Date presented were repeated for at least three times (biological replicates) and the successful attempts at replication were confirmed.
Randomization	Randomization did not apply to this research as it did not involve animal experiments and clinical trials.
Blinding	Blinding did not apply to this research as it did not involve animal experiments and clinical trials.

Reporting for specific materials, systems and methods

We require information from authors about some types of materials, experimental systems and methods used in many studies. Here, indicate whether each material, system or method listed is relevant to your study. If you are not sure if a list item applies to your research, read the appropriate section before selecting a response.

Materials & experimental systems

n/a	Involved in the study	
☐	☒	Antibodies
☐	☒	Eukaryotic cell lines
☒	☐	Palaeontology and archaeology
☒	☐	Animals and other organisms
☒	☐	Human research participants
☒	☐	Clinical data
☒	☐	Dual use research of concern

Methods

n/a	Involved in the study	
☒	☐	ChIP-seq
☒	☐	Flow cytometry
☒	☐	MRI-based neuroimaging

Antibodies

Antibodies used	Alexa Fluor 488-phalloidin, Alexa Fluor 594-goat anti-rabbit IgG, HRP-conjugated goat anti-rabbit IgG, and HRP-conjugated goat anti-mouse IgG were purchased from Thermo Fisher Scientific (Waltham, MA, USA). Anti-β-catenin (D10A8), anti-phospho-β-catenin (pSer41), anti-Axin1 (C76H11), anti-GSK-3β, anti-phospho-GSK-3β (pSer9), anti-cyclin D1, and anti-p21 antibodies were from Cell Signaling Technology (Beverly, MA, USA). The anti-8-OHdG (15A3) antibody was from Santa Cruz Biotechnology (Dallas, TX, USA) and anti-β-tubulin was purchased from Sigma Aldrich (St Louis, Mo, USA). The anti-ApoF (ab231585) antibody was from Abcam (Cambridge, MA, USA).
Validation	The staining of the antibody is evaluated by Western blot through analyses of samples from NSF and MSF cell lysates.

Eukaryotic cell lines

Policy information about cell lines

Cell line source(s)	Primary adult NMR Skin/Lung Fibroblasts, Primary adult Mouse Skin Fibroblasts, NIH3T3
Authentication	Primary NMR cell lines were authenticated by Department of Aging and Longevity Research, Faculty of Life Sciences, Kumamoto University. The data that support the cell line authentication are openly available at https://doi.org/10.1038/ncomms11471. NIH3T3 were purchased from ATCC.
Mycoplasma contamination	All cell lines tested negative for mycoplasma contamination
Commonly misidentified lines (See ICLAC register)	None
